# The performance of using dried blood spot specimens for HIV-1 viral load testing: A systematic review and meta-analysis

**DOI:** 10.1371/journal.pmed.1004076

**Published:** 2022-08-22

**Authors:** Lara Vojnov, Sergio Carmona, Clement Zeh, Jessica Markby, Debrah Boeras, Marta R. Prescott, Anthony L. H. Mayne, Souleymane Sawadogo, Christiane Adje-Toure, Guoqing Zhang, Mercedes Perez Gonzalez, Wendy S. Stevens, Meg Doherty, Chunfu Yang, Heather Alexander, Trevor F. Peter, John Nkengasong, the DBS for VL Diagnostics Investigation Consortium

**Affiliations:** 1 Clinton Health Access Initiative, Boston, Massachusetts, United States of America; 2 National Health Laboratory Service, Johannesburg, South Africa; 3 Center for Global Health, Division of Global HIV/TB, US Centers for Disease Control, Atlanta, Georgia, United States of America; 4 World Health Organization, Geneva, Switzerland; 5 University of South Africa, Johannesburg, South Africa; 6 Center for Global Health, Division of Global HIV/TB, US Centers for Disease Control, Windhoek, Namibia; 7 Center for Global Health, Division of Global HIV/TB, US Centers for Disease Control, Abidjan, Cote d’Ivoire; 8 Department of Molecular Medicine and Haematology, University of the Witwatersrand, Johannesburg, South Africa; University of Southampton, UNITED KINGDOM

## Abstract

**Background:**

Accurate routine HIV viral load testing is essential for assessing the efficacy of antiretroviral treatment (ART) regimens and the emergence of drug resistance. While the use of plasma specimens is the standard for viral load testing, its use is restricted by the limited ambient temperature stability of viral load biomarkers in whole blood and plasma during storage and transportation and the limited cold chain available between many health care facilities in resource-limited settings. Alternative specimen types and technologies, such as dried blood spots, may address these issues and increase access to viral load testing; however, their technical performance is unclear. To address this, we conducted a meta-analysis comparing viral load results from paired dried blood spot and plasma specimens analyzed with commonly used viral load testing technologies.

**Methods and findings:**

Standard databases, conferences, and gray literature were searched in 2013 and 2018. Nearly all studies identified (60) were conducted between 2007 and 2018. Data from 40 of the 60 studies were included in the meta-analysis, which accounted for a total of 10,871 paired dried blood spot:plasma data points. We used random effects models to determine the bias, accuracy, precision, and misclassification for each viral load technology and to account for between-study variation. Dried blood spot specimens produced consistently higher mean viral loads across all technologies when compared to plasma specimens. However, when used to identify treatment failure, each technology compared best to plasma at a threshold of 1,000 copies/ml, the present World Health Organization recommended treatment failure threshold. Some heterogeneity existed between technologies; however, 5 technologies had a sensitivity greater than 95%. Furthermore, 5 technologies had a specificity greater than 85% yet 2 technologies had a specificity less than 60% using a treatment failure threshold of 1,000 copies/ml. The study’s main limitation was the direct applicability of findings as nearly all studies to date used dried blood spot samples prepared in laboratories using precision pipetting that resulted in consistent input volumes.

**Conclusions:**

This analysis provides evidence to support the implementation and scale-up of dried blood spot specimens for viral load testing using the same 1,000 copies/ml treatment failure threshold as used with plasma specimens. This may support improved access to viral load testing in resource-limited settings lacking the required infrastructure and cold chain storage for testing with plasma specimens.

## Abstract 2

Lara Vojnov and co-workers report on the use of dried blood spots for HIV viral load testing.

## Author summary

**Why was this study done?:**

- Though the preferred method to monitor patients on antiretroviral treatment (ART), there remains limited access to viral load testing, particularly in resource-limited settings suffering from infrastructural challenges.
- Dried blood spots can be an alternative sample type for viral load testing in resource-limited settings as storage requirements are do not require cold chain or rapid transportation to the laboratory.
- Several viral load technologies exist with regulatory approvals for use with plasma samples; however, a significant knowledge gap exists in the accuracy of using dried blood spot samples for HIV viral load testing.

**What did the researchers do and find?:**

- We conducted a systematic review and meta-analysis and identified 60 studies that fulfilled the inclusion criteria and included data on 6 technologies.
- Though some heterogeneity was observed in the systematic review, the meta-analysis including 10,871 data points from 40 studies highlighted clear clinical performance metrics for each technology. Four of the 6 technologies had a sensitivity and specificity above 83% using dried blood spot samples for viral load testing with a treatment failure threshold of 1,000 copies/ml.
- Most studies used dried blood spots prepared in the laboratory with precision pipetting resulting in consistent measurement of blood applied to each spot. Additional studies would be useful to ensure the results observed can be replicated and remain consistent in the field when utilizing other sample preparation techniques.

**What do these findings mean?:**

- Dried blood spot samples can be used for viral load testing at a treatment failure threshold similar to plasma samples at 1,000 copies/ml.
- Performance of dried blood spot samples is not perfect; therefore, some misclassification will occur. A small proportion of patients successfully undetectable will be incorrectly considered failing, while a small proportion of patients failing treatment will be considered successfully treated. Furthermore, selection and implementation of technologies for use with dried blood spots may be complicated with heterogenous performance and regulatory approvals.
- The availability and use of alternative sample types, such as dried blood spots, that require less stringent and robust infrastructure may allow for significant expansion of access to viral load testing in resource-limited settings.

## Introduction

Although an estimated nearly 37 million HIV–positive people are currently eligible for antiretroviral treatment (ART) according to the 2016 WHO Consolidated guidelines on the use of antiretroviral drugs for treating and preventing HIV infection, only 19.5 million people are on ART worldwide [1]. The global community is focused on the goal of achieving universal access to affordable and effective ART with the intention of moving toward elimination of HIV infection. To address this gap, UNAIDS released global HIV targets of 90-90-90, in which the third 90 target represents achieving viral suppression in 90% of those receiving ART. Suppressed or undetectable viral loads improve the health of patients as well as significantly reduce the likelihood of HIV transmission [2–5]. To reach these goals, there is substantial impetus to ensure HIV–positive people receive high-quality care, which includes accurate monitoring of viral load.

Accurate routine viral load testing is essential for assessing the efficacy of ART regimens in preventing morbidity, mortality, and transmission as well as treatment adherence and is an indicator of the potential emergence of drug resistance [2,6–13]. Routine viral load testing has also been strongly recommended by WHO as the preferred method for monitoring patients on ART based on evidence indicating that viral load detects ART failure earlier compared to clinical and immunological assessments [14–18]. Testing using plasma separated from EDTA anti-coagulated whole blood has been used in developed countries for many years as the gold standard for treatment monitoring [8,9,11,13]. Unfortunately, the high costs of viral load equipment, the requirement for highly specialized and well-equipped laboratories, and particularly the difficulty of fresh blood specimen collection, storage, and transportation logistics have restricted testing to centralized laboratories and slowed the scale-up of viral load testing in resource-limited countries.

Though viral load testing using plasma is the gold standard, alternative specimen types may enable expansion of access to viral load testing in resource-limited countries. Dried blood spot specimens for HIV testing are well established in resource-limited settings and have been routinely used for collecting and shipping early infant HIV diagnosis specimens for testing by polymerase chain reaction in centralized laboratories. They are beneficial as they do not require centrifuges, refrigerators, or freezers at the specimen collection site, can be stored and transported for weeks at ambient temperature, and require a simple finger-prick or heel-stick blood specimen that can be prepared by lower cadres of health care facility staff. Similar benefits could be achieved by using dried blood spot specimens for viral load testing programs in resource-limited settings.

Dried blood spot specimens for viral load testing using nucleic acid-based detection methods utilize whole blood as the input specimen, which can result in extraction and detection of intracellular HIV proviral DNA and cell-associated HIV RNA in addition to the biomarker target of free viral RNA circulating in the plasma. Due to this potential over-quantification of HIV nucleic acids using dried blood spot specimens, the 2013 WHO Consolidated ART Guidelines recommended a higher threshold of 3,000 to 5,000 copies/ml to identify treatment failure using dried blood spot specimens, while maintaining a threshold of 1,000 copies/ml to identify treatment failure with plasma specimens [18]. The uncertainty of the performance of dried blood spot specimens for viral load testing has triggered significant interest and controversy as countries are contemplating the scale-up of viral load testing using dried blood spot specimens. Individual studies conducted to date comparing the performance of dried blood spot specimens to plasma on viral load technologies have not provided a consistent picture of performance to inform testing policy [19–69]. Additionally, differences in analytical approaches between studies have made it difficult to compare the data on key criteria such as clinical misclassification and dried blood spot specimen performance at lower thresholds as these have not been presented consistently. This study is a systematic review and meta-analysis using primary data from studies reporting on the currently available viral load technologies and a standardized analysis methodology to better understand the performance and limitations of this specimen collection type across all commonly used viral load testing platforms.

## Materials and methods

### Search strategy

**Fig 1:**
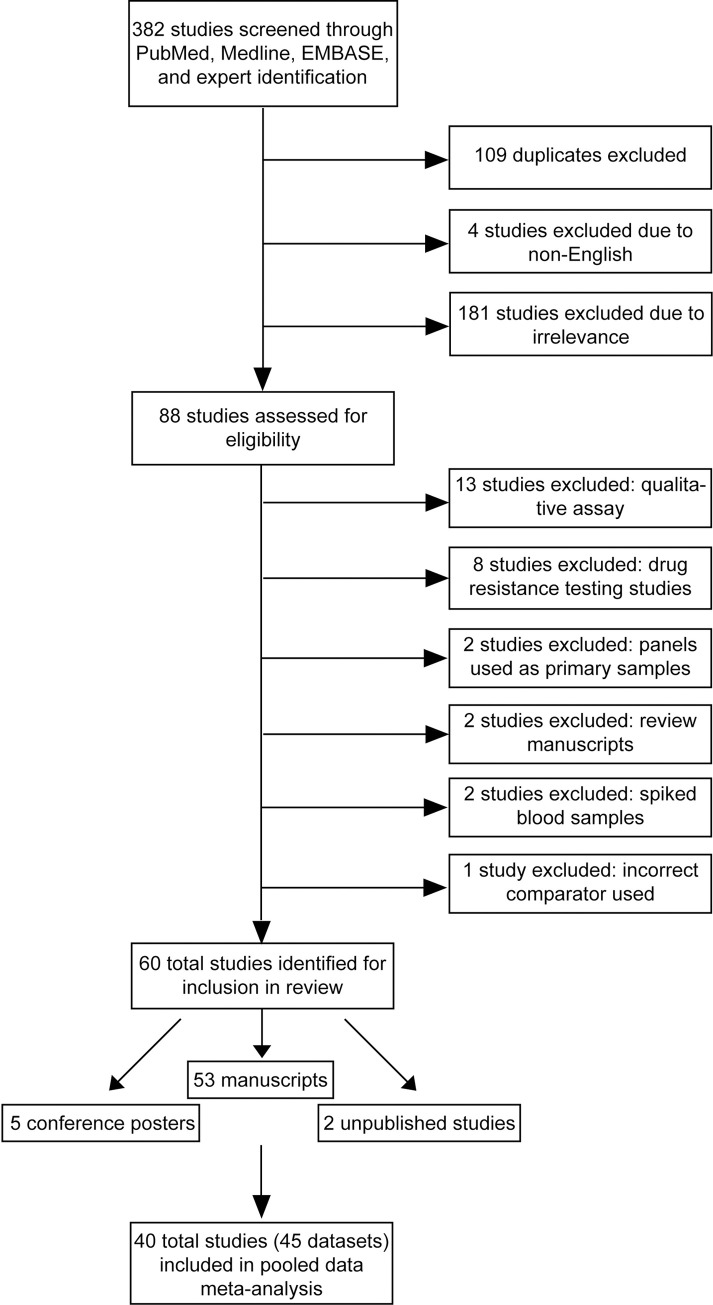
PRISMA flow chart.

Fig 1 shows a PRISMA (Preferred Reporting Items for Systematic Reviews and Meta-Analyses) diagram demonstrating the study selection and data acquisition process [70]. An initial search was conducted on May 29, 2013 in the PubMed, EMBASE, Google Scholar, and Medline databases to identify peer-reviewed original research with appropriate data for this systematic review and meta-analysis. Conference abstracts within the search dates from the Conference on Retroviruses and Opportunistic Infections (CROI), International Conference on AIDS and STIs in Africa (ICASA), International AIDS Society (IAS), and AIDS Conferences as well as extensive bibliography and gray literature were screened for possible inclusion. Only English titles and manuscripts were included. A full final search was conducted again on April 20, 2018 (S1 Fig). For inclusion, studies must have compared viral load values using dried blood spot and plasma specimens measured by 1 or more of the following 6 commonly used technologies—Abbott RealTi*m*e HIV-1 on the m2000 platform (Abbott Molecular, Abbott Park, Illinois, United States of America), Generic HIV Charge Virale (Biocentric, Bandol, France), bioMérieux NucliSENS EasyQ HIV-1 v2.0 (bioMérieux, Craponne, France), Hologic Aptima (Hologic, Marlborough, Massachusetts, USA), Roche Amplicor HIV-1 Monitor Test, v1.5 or COBAS Ampliprep/COBAS TaqMan HIV-1 Test, v2.0 using both specimen preextraction reagent (SPEX) and free virus elution (FVE) dried blood spot specimen protocols (Roche Molecular Systems, Basel, Switzerland), and Siemens VERSANT HIV-1 RNA 1.0 assay (kPCR) (Siemens Healthcare Diagnostics, Munich, Germany). Search terms included “dried blood spot,” “plasma,” “technical performance,” “comparison,” “evaluation,” “viral load testing,” and each of the proprietary respective technology names.

### Study selection

Studies were included if they included technical evaluation data comparing dried blood spot samples to plasma, were pertaining to viral load testing, and were performed using HIV–positive blood. Studies were excluded if they used spike blood samples or panels, compared dried blood spot samples to plasma with a different assay, performed a qualitative analysis of dried blood spot samples, or the comparator was a sample type other than plasma. Sixty studies were identified through online searches and expert notification (Fig 1). We contacted the corresponding authors of all studies that met the inclusion criteria to explain the analysis plan and request original data and obtained original data from 4 studies that were not yet published. For the meta-analysis, a total of 40 studies provided 45 data sets across the 6 technologies resulting in a total of 10,831 paired dried blood spot and plasma viral load results. Correction factors were applied as suggested by the manufacturers for the Hologic Aptima and Roche COBAS TaqMan FVE technologies. Due to the discontinuation of the Roche Amplicor HIV-1 Monitor test, v1.5, data using this technology were excluded from the analysis. Study characteristics were extracted from each manuscript or through author contact.

### Quality assessment

The Standards for Reporting Studies of Diagnostic Accuracy (STARD) criteria and Quality Assessment of Diagnostic Accuracy Studies (QUADAS-2) were followed and each study graded for quality [71,72]. The PRISMA reporting guideline was followed (S1 PRISMA Checklist).

### Statistical analyses

All analyses performed were prespecified in the protocol. Study variables analyzed for each study included study sample size, viral load mean and median, proportion of patient specimens within specific viral load ranges, and sensitivity and specificity. Sensitivity was calculated as the proportion of dried blood spot specimens correctly identified as failing or above the defined treatment failure threshold. Specificity was calculated as the proportion of dried blood spot specimens correctly identified as not failing or below the treatment failure threshold. Forest plots were developed to analyze the between-study heterogeneity of diagnostic performance for each technology. Primary data were then pooled to analyze the performance of dried blood spot specimens for each technology. The median pooled viral load was calculated accounting for between-study heterogeneity using a random effects model. Viral load values were log-transformed because of the non-normal distribution of the data.

Patients with 2 consecutive viral loads 3 months apart above 1,000 copies/ml with adherence counseling after the first viral load are to be considered failing their ART regimen [18]. The performance (sensitivity, specificity, and clinical misclassification) of dried blood spot specimens compared with plasma on each platform was assessed. Since longitudinal data on dried blood spot specimen performance were not available, cross-sectional comparisons were performed. In addition, alternate treatment failure thresholds for viral load using dried blood spot specimens were assessed including 1,000, 3,000, 5,000, 7,500, and 10,000 copies/ml to understand the threshold that best compares to the gold standard plasma specimens for each technology. Each treatment failure threshold of dried blood spot specimens was compared to 1,000 copies/ml for plasma with measurements of true positives, true negatives, false positives, and false negatives calculated for each technology to create pooled estimates of diagnostic accuracy of dried blood spot specimens for each platform across all studies. Using these treatment failure thresholds, the sensitivity, specificity, upward and downward misclassification rates, and positive and negative predictive values were also calculated. Misclassification was calculated as the proportion of dried blood spot specimens incorrectly identified as above or below 1,000 copies/ml compared to the plasma specimens. Upward misclassification was defined as the number of dried blood spot specimens incorrectly identified as above the tested treatment failure threshold divided by the total number of matched plasma specimens with viral load results below 1,000 copies/ml. Downward misclassification was defined as the number of dried blood spot specimens incorrectly identified as below the tested treatment failure threshold divided by the total number of matched plasma specimens with viral load results above 1,000 copies/ml.

Pooled estimates of accuracy and misclassification were calculated using a series of methods to first quantify and then account for the presence of study heterogeneity when calculating pooled estimates. To determine the presence of between-study heterogeneity, the Q-statistic was calculated for each treatment failure threshold comparison (for example, 1,000 copies/ml using dried blood spot specimens to 1,000 copies/ml using plasma specimens; 3,000 copies/ml using dried blood spot specimens to 1,000 copies/ml using plasma specimens, etc.) [73]. After confirming the presence of heterogeneity, random effects models were used to estimate the pooled summary measures for bias, accuracy, and fit hierarchical summary receiver-operator curves (HSROCs) accounting for between-study variation. For sensitivity and specificity values and corresponding 95% confidence intervals, bivariate random effects modeling was used to simultaneously determine the pooled estimates, accounting for the covariance of sensitivity and specificity as well as study specific heterogeneity [74]. Univariate random effects models were used when less than 4 studies were included in the model methods, as bivariate random effects models were unstable. To obtain pooled estimates of misclassification, univariate random effects models were used to obtain the point estimates and corresponding 95% confidence intervals [75]. All statistics were calculated overall, for each technology, and for each study data set. We included a continuity correction to all of the diagnostic values of a study if at least 1 diagnostic value had a zero value.

Bland–Altman plots were created to assess mean bias values. In these plots, the mean viral load value was assessed by dried blood spot specimens and plasma (x axis) compared to the mean difference between paired dried blood spot specimens and plasma specimens (y axis) to evaluate the degree of agreement between the 2 methods.

Several sub-analyses were conducted to further examine the diagnostic accuracy and clinical misclassification of dried blood spot specimens by specific populations or technologies. First, the diagnostic accuracy statistics were calculated for the comparison of 1,000 copies/ml using dried blood spot specimens to 1,000 copies/ml using plasma specimens limiting the analysis to studies that included only patients on antiretroviral therapy. Second, the diagnostic accuracy statistics were measured stratifying those studies that processed dried blood spot specimens and performed viral load testing according to the manufacturer’s recommended protocol and those that did not. Additionally, sub-analyses were analyzed by specimen collection method (capillary versus venous blood collection), specimen storage method (fresh versus frozen), geography, and dried blood spot card type. Finally, in a third sub-analysis only data from the most recent version of the specified technologies were included.

Three researchers independently performed the statistical analysis to ensure accuracy. Graphic representations were completed in GraphPad Prism (La Jolla, California, USA) and analyses were completed in Stata 13 (College Station, Texas, USA) and SAS version 9.2 (Cary, North Carolina, USA).

### Protocol

The prepared protocol was reviewed by the World Health Organization and approved by Chesapeake Institutional Research Review Board (Columbia, Maryland, USA; www.chesapeakeirb.com) (S1 Text).

## Results

### Systematic review

A total of 60 studies were identified for inclusion (Fig 1 and Table 1) [19–69]. Thirteen studies were excluded due to using a qualitative assay, 8 studies were drug resistance testing studies, 2 studies used panels as primary sample types, 2 studies were review manuscripts lacking primary data, 2 studies used spike blood samples, and 1 study used an incorrect comparator. There was low to moderate heterogeneity in the analytical and clinical performance comparisons within technologies as well as in the viral load medians and distributions (Table 2).

**Table 1:** Study characteristics and dried blood spot specimen preparation process.

First author	Reference	Manuscript date	Technology	DBS paper type	vDBS blood type/volume	DBS drying time	Number spots used	DBS storage	DBS prep: buffer/mix time	Sample/NAT isolation	Location	Sample size	Venous or finger-prick	All patients on ART?
Abravaya	[19]	2008	Abbott m2000rt RealTime HIV-1	Whatman 903	50 ul	Unknown	2	Unknown	1.7 ml Abbott lysis buffer/2 h	Abbott m2000sp	Unknown	NR	Venous	Unknown
Arredondo	[23]	March 2011	Abbott m2000rt RealTime HIV-1	Whatman 903	50 ul	4–6 h at RT	2	Bag with silica -20C	2 ml Abbott lysis buffer/2 h	Abbott m2000sp	Spain	157	Venous	30%
Carmona	[26]	Unknown	Abbott m2000rt RealTime HIV-1	Munktell TFN	70 ul EDTA	O/N at RT	2	Bag at RT	1.7 ml Abbott lysis buffer/30 min	Abbott m2000sp	South Africa	113	Venous	Unknown
David	[27]	June 2012	Abbott m2000rt RealTime HIV-1	Whatman 903	50 ul EDTA	6–8 h at RT	2	Bag with desiccant -20C	1.7 ml Abbott lysis buffer/2 h	Abbott m2000sp	India	62	Venous	Unknown
Erba	[28]	Dec 2015	Abbott m2000rt RealTime HIV-1	Unknown	50 ul EDTA	O/N at RT	2	Bag with desiccant at RT	1.7 ml Abbott lysis buffer/15 min	Abbott m2000sp	Malawi and Mozambique	277	Venous	Unknown
Garrido	[31]	Nov 2008	Abbott m2000rt RealTime HIV-1	Whatman 903	50 ul EDTA	O/N at RT	1	4C until processed	2 ml NucliSens lysis buffer/2 h	Abbott m2000sp	Spain	81	Venous	Unknown
Lofgren	[37]	Nov 2009	Abbott m2000rt RealTime HIV-1	Whatman 903	50 ul EDTA	>4 h RT	2	Bag with desiccant	1.7 ml Abbott lysis buffer/2 h	Abbott m2000sp	Tanzania	313	Venous	53%
Marconi	[38]	June 2008	Abbott m2000rt RealTime HIV-1	Whatman 903	50 ul	1–170 days at RT	2	Unknown	2 ml Abbott lysis buffer/ 2 h	Abbott m2000sp	Italy	163	Venous	Unknown
Mbida	[39]	Dec 2008	Abbott m2000rt RealTime HIV-1	Whatman 903	50 ul	O/N at RT	2	Bag at RT	1.7 ml Abbott lysis buffer/2 h	Abbott m2000sp	Cameroon; DBS France	45	Venous	12 of 27
Monleau	[42]	Dec 2013	Abbott m2000rt RealTime HIV-1	Whatman 903	50 ul EDTA	3 h at RT	2	Silica 2–4 weeks RT, -20C	1.7 ml Abbott lysis buffer/30 min		Africa, Asia	173	Venous	Yes, >24 m
Mwau		Unknown	Abbott m2000rt RealTime HIV-1	Unknown							Kenya	216	Venous	
Neogi	[45]	Nov 2011	Abbott m2000rt RealTime HIV-1	Whatman 903	50 ul EDTA	O/N at RT	2	Bag with silica RT	1.7 ml Abbott lysis buffer/2 h	Abbott m2000sp	India	125	Venous	71 of 125
Rutstein	[54]	May 2014	Abbott m2000rt RealTime HIV-1	Munktell TFN	50 ul	3 h at RT	2	Bag with desiccant at RT	1.7 ml Abbott lysis buffer/30 min	Abbott m2000sp	Malawi	547	Venous	Yes
Schmitz	[57]	April 2017	Abbott m2000rt RealTime HIV-1	Whatman 903	50 ul EDTA	O/N at RT	2	Bag with desiccant at RT	Abbott lysis buffer	Abbott m2000sp	Kenya	416	Both	Most
Taieb	[58]	2018	Abbott m2000rt RealTime HIV-1	Munktell TFN	70 ul EDTA	3 h at RT	1	Bag with desiccant -20C	1.3 ml Abbott lysis buffer/30 min	Abbott m2000sp	Vietnam	401	Venous	76%
Tang	[59]	2017	Abbott m2000rt RealTime HIV-1	Munktell TFN	70 ul	Unknown	1	RT or -20C	1.3 ml Abbott lysis buffer/30 min	Abbott m2000sp	Cote d’Ivoire, Uganda, South Africa	497	Both	49%
Vidya	[63]	Oct 2011	Abbott m2000rt RealTime HIV-1	Whatman 903	50 ul	O/N at RT	2	Bag with silica RT	1.7 ml Abbott lysis buffer/2 h	Abbott m2000sp	India	100	Venous	Unknown
Zeh	[69]	2017	Abbott m2000rt RealTime HIV-1	Whatman 903	50 ul	O/N at RT	2	Bag at 2-8C	1.7 ml Abbott lysis buffer/30 min	Abbott m2000sp	Kenya	200	Venous	Yes
Monleau		Jan 2010	Biocentric G2 Generic	Whatman 903	50 ul	3 h at RT	4	Bag with silica -20C	4 ml NucliSens lysis buffer/2 h	NucliSens miniMAG	France	34	Venous	Unknown
Monleau	[42]	Dec 2013	Biocentric G2 Generic	Whatman 903	50 ul EDTA	3 h at RT	2	Silica 2–4 weeks RT, -20C	2 ml NucliSens lysis buffer/30 min	NucliSens miniMAG	Africa, Asia	118	Venous	Yes, >24 m
Reigadas	[52]	Feb 2009	Biocentric	Whatman 903	50 ul EDTA	1 h at RT	5	Bag with desiccant at 4C	220 ul lysis buffer/1 h, QIAamp	QIAamp	France	86	Venous	17
Viljoen	[64]	Jan 2010	Biocentric	Whatman 903	50 ul EDTA	O/N at RT	1 or 2	Bag with silica -20C	1: 9 ml NucliSens/1 h 2: 2ml NucliSens/1 h	NucliSens miniMAG	South Africa, Burkina Faso	327	Venous	Some
Yapo	[67]	July 2013	Biocentric	Whatman 903	Unknown	Unknown	3	-80C	180 ul buffer/1 h	QIAamp	Cote d’Ivoire	138	Venous	None
Alvarez-Munoz	[21]	Sep 2004	bioMerieux NucliSens HIV-1 QT	Whatman 903	50 ul EDTA	O/N at RT	2	Bag with silica RT	9 ml NucliSens lysis buffer/2 h	NucliSens extraction	Mexico	124	Venous	Unknown
Ayele	[24]	Sep 2006	bioMerieux NucliSens HIV-1 QT	Whatman 903	50 ul EDTA	Unknown	4	Bag with desiccant at RT	3 ml lysis buffer/2 h or O/N	NucliSens extraction/Boom method	Ethiopia	19	Venous	Unknown
Brambilla	[25]	July 2002	bioMerieux NucliSens HIV-1 QT	Whatman 903	50 ul	O/N at RT	Unknown	Bag with silica -20C	9 ml NucliSens lysis buffer/2 h	NucliSens extraction/Boom method	Unknown	76	Venous	Unknown
Fajardo	[29]	Feb 2014	bioMerieux NucliSens EasyQ HIV-1 v2.0	Whatman 903	50 ul	O/N at RT	2	Bag with desiccant at RT	2 ml NucliSens lysis buffer/30 min	NucliSens easyMAG	Malawi	770	Venous	Some
Fiscus	[30]	May 1997	bioMerieux NucliSens HIV-1 QT	Whatman 903	50 ul/100 ul EDTA	O/N at RT	2 of 50 ul each	RT until processed	9 ml NucliSens lysis buffer/2 h	NucliSens extraction	USA	76	Venous	Unknown
Garrido	[31]	Nov 2008	bioMerieux NucliSens EasyQ HIV-1 v1.1	Whatman 903	50 ul EDTA	O/N at RT	1	4C until processed	2 ml NucliSens lysis buffer/2 h	NucliSens extraction/Boom method	Spain	97	Venous	Unknown
Johannessen	[33]	Jan 2009	bioMerieux NucliSens EasyQ HIV-1 v1.2	Whatman 903	PPT:DBS, plasma	O/N at RT	2	Bag with silica -20C	9 ml NucliSens lysis buffer/2 h	NucliSens extraction/Boom method	Tanzania	98	Venous	Yes, >6 months
Lira	[36]	Nov 2009	bioMerieux NucliSens HIV-1 QT	Whatman 903	50 ul EDTA	4 h at RT	2	Bag with silica -70C	9 ml NucliSens lysis buffer/2 h	NucliSens extraction	Mexico	57	Venous	84.50%
Mercier-Delarue	[40]	Oct 2013	bioMerieux NucliSens EasyQ HIV-1 v2.0	Whatman 903	50 ul EDTA	RT	2	Bag at RT	2 ml NucliSens lysis buffer/2 h	NucliSens easyMAG	Niger	192	Venous	Some
Monleau	[42]	Dec 2013	bioMerieux NucliSens EasyQ HIV-1 v1.2	Whatman 903	50 ul EDTA	3 h at RT	2	Silica 2–4 weeks RT, -20C	2 ml NucliSens lysis buffer/30 min	NucliSens miniMAG	Africa, Asia	91	Venous	Yes, >24 m
Mwaba	[44]	Dec 2003	bioMerieux NucliSens HIV-1 QT?	Whatman 903	50 ul	3 h at RT	Unknown	Bag with silica RT	9 ml NucliSens lysis buffer/2 h	NucliSens extraction/Boom method	Zambia; DBS Canada	51	Venous	None
Rottinghaus	[53]	Oct 2011	bioMerieux NucliSens EasyQ HIV-1 v1.1	Whatman 903	100 ul EDTA	O/N at RT	1	Bag with desiccant at RT	2 ml NucliSens lysis buffer/30 min	NucliSens easyMAG	Nigeria; DBS Atlanta	173	Venous	Yes
Toure Kane	[34]	Apr 2007	bioMerieux NucliSens EasyQ HIV-1 v1.2	Whatman 903	50 ul EDTA	O/N at RT	2	Bag with silica RT	2 ml Abbott lysis buffer/30 min	NucliSens miniMAG/Boom method	France, Senegal	41	Venous	Unknown
Uttayamakul	[61]	Jan 2005	bioMerieux NucliSens HIV-1 QT	Whatman 903	50 ul EDTA	O/N at RT	1	Bag with desiccant -20C	1 ml lysis buffer/30 min	NucliSens extraction/Boom method	Thailand	209	Venous	No
van Deursen	[62]	July 2009	bioMerieux NucliSens EasyQ HIV-1 v2.0	Whatman 903	50 ul EDTA	3 h—O/N at RT	2	Bag with desiccant at RT	2 ml Abbott lysis buffer/30 min	NucliSens easyMAG	Netherlands and France	218	Venous	Yes
Gous		Oct 2016	Hologic Aptima HIV-1 Quant Dx	Whatman 903	70 ul EDTA	O/N at RT	1	Bag with desiccant -70C	1 ml Hologic buffer for 30 at RT	Integrated into Aptima Panther	South Africa	20	Venous	Some
Sahoo	[55]	Aug 2016	Hologic Aptima HIV-1 Quant Dx	Whatman 903	50 ul EDTA	Unknown	1	-80C	1 ml Hologic buffer for 30 at RT	Integrated into Aptima Panther	USA	162	Venous	Unknown
Yek	[68]	Aug 2017	Hologic Aptima HIV-1 Quant Dx	Whatman 903	50 ul EDTA	O/N at RT	1	Bag with desiccant at RT or -20C	1 ml Hologic buffer for 30 at RT	Integrated into Aptima Panther	USA	104	Venous	Some
Aitken	[20]	Jan 2013	Roche COBAS Ampliprep/TaqMan v2	Unknown	50 ul EDTA	Unknown	2	Varied	700 ul elution buffer, QIAamp	Roche COBAS Ampliprep	Uganda	409	Venous	Unknown
Andreotti	[22]	Aug 2009	Roche COBAS TaqMan RT-PCR	Whatman 903	75 ul EDTA	4 h at RT	1	Bag with desiccant -20C	2 ml NucliSens lysis buffer/O/N	NucliSens miniMAG/Boom method	Malawi; DBS Italy	129	Venous	18
Brambilla	[25]	July 2002	Roche Amplicor Monitor v1.5	Whatman 903	50 ul	O/N at RT	Unknown	Bag with silica -20C	0.75 ml CORD solution/30 min	Monitor specimen diluent	Unknown	3	Venous	Unknown
Carmona	[26]	2011	Roche COBAS Ampliprep/TaqMan v2	Munktell TFN	70 ul EDTA	3 h at RT	1	Bag at RT	1 ml SPEX/10 min at 56C	Roche COBAS Ampliprep	South Africa	107	Venous	Unknown
Carmona		Unknown	Roche COBAS Ampliprep/TaqMan v2	Munktell TFN	70 ul EDTA	O/N at RT	1	Bag at RT	1 ml PBS/1 h at RT	Roche COBAS Ampliprep	South Africa	281	Venous	Unknown
Ikomey	[32]	Unknown	Roche Amplicor Monitor v1.5	Whatman 903	50 ul EDTA	2 h—O/N at RT	4	Bag	900 ul lysis buffer/cen 20 m	Roche	Cameroon	60	Venous	Unknown
Leelawiwat	[35]	July 2008	Roche Amplicor Monitor v1.5	Whatman 903	≈50 ul heel-prick	24–48 h at RT	1	Bag with desiccant	0.9 ml NucliSens lysis buffer/2 h	NucliSens extraction	Thailand	56	Venous	Mono or dual therapy
Mercier-Delarue	[40]	Oct 2013	Roche COBAS Ampliprep/TaqMan v2	Whatman 903	50 ul EDTA	RT	2	Bag at RT	2 ml NucliSens lysis buffer/2 h	Roche COBAS Ampliprep	Niger	197	Venous	Some
Onkendi	[47]	2017	Roche COBAS Ampliprep/TaqMan v2	Whatman 903	70 ul EDTA	4 h at RT	1	Bag with desiccant	1 ml PBS/1 h at RT	Roche COBAS Ampliprep	Kenya	180	Venous	Some
Ouma	[48]	Nov 2012	Roche COBAS Amplicor v1.5/TaqMan v2	Whatman 903	75 ul EDTA	O/N at RT	1	Bag with desiccant -20C	600 ul Qiagen elution buffer/15 min at 56C	Roche COBAS Ampliprep	Kenya	432	Venous	No
Pannus	[49]	2016	Roche COBAS Ampliprep/TaqMan v2	Whatman 903	70 ul EDTA	O/N at RT	1	Bag with desiccant -20C	1 ml PBS/1 h at RT	Roche COBAS Ampliprep	Belgium	261	Venous	Unknown
Pollack	[51]	2018	Roche COBAS Ampliprep/TaqMan v2	Whatman 903	70 ul	4 h at RT	1	Bag with desiccant -4C	1 ml SPEX/10 min at 56C and 1 ml PBS/1 h at RT	Roche COBAS Ampliprep	Vietnam	876	Venous	Some
Sawadogo	[56]	Aug 2014	Roche COBAS Amplicor v1.5/TaqMan v2	Whatman 903	50 ul EDTA	Unknown	1	Bag with desiccant -70C	1.1 ml SPEX/10min	Roche COBAS Ampliprep	Namibia	823	Venous	Yes, >6 months
Tariro Makadzange	[60]	May 2017	Roche COBAS Ampliprep/TaqMan v2	Whatman 903	50 ul	O/N at RT	1	Bag with dessicant at RT	1 ml PBS/1 h at RT	Roche COBAS Ampliprep	Zimbabwe	272	Venous	Unknown
Waters	[65]	Feb 2007	Roche COBAS TaqMan RT-PCR	Whatman 903	Not indicated	RT	Unknown	Bag at RT	Unknown	Primagen	Uganda; DBS Europe	307	Venous	Yes
Wu	[66]	Unknown	Roche COBAS Ampliprep/TaqMan v2	Whatman 903	70 ul	Unknown	1	Unknown	1 ml PBS/30 min	Roche COBAS Ampliprep	USA	196	Venous	Unknown
Zeh		Unknown	Roche COBAS Ampliprep/TaqMan v2	Whatman 903	50 ul	O/N at RT	1	Bag at 2–8C	1.1 ml SPEX/10 min	Roche COBAS Ampliprep	Kenya	200	Venous	Yes
Zeh		Unknown	Roche COBAS Ampliprep/TaqMan v2	Whatman 903	50 ul	O/N at RT	1	Bag at 2–8C	1.1 ml PBS/30 min	Roche COBAS Ampliprep	Kenya	171	Venous	Yes
Zhang		Unknown	Roche COBAS Ampliprep/TaqMan v2	Whatman 903	70 ul EDTA	O/N at RT	1	Bag at RT	1 ml PBS/1 h at RT	Roche COBAS Ampliprep	Cote d’Ivoire	1093	Venous	Yes
Zinyowera	[43]	2013	Roche COBAS TaqMan RT-PCR	Munktell TFN	70 ul finger-prick	3 h at RT	1	Unknown	1 ml SPEX/10 min at 56C	Roche COBAS Ampliprep	Zimbabwe	119	Finger-prick	Yes
Pirollo	[50]	May 2011	Siemens VERSANT HIV-1 RNA 1.0 (kPCR)	Whatman 903	50 ul EDTA	RT	1	Bag with desiccant at RT	1.25 Siemens lysis buffer	VERSANT Sample Preparation Module	Italy	98	Venous	Unknown
Siemens			Siemens VERSANT HIV-1 RNA 1.0 (kPCR)	Unknown								46	Venous	

**Table 2:** Analytical and clinical metrics for each study.

		Reference	R^2^	bias (95% CI)	Median plasma	Median DBS	Difference in medians	Proportion undetectable	Proportion 1–10,000 cp/ml	Proportion > 10,000 cp/ml
Abravaya	Abbott m2000rt RealTime HIV-1	[19]	Quantitative data not reported							
Aitken	Roche COBAS Ampliprep/TaqMan v2	[20]		NR						
Alvarez-Munoz	bioMerieux NucliSens HIV-1 QT	[21]	0.9025	NR						
Andreotti	Roche COBAS TaqMan RT-PCR	[22]	0.8328	−0.474 (−1.98–1.03)	4.07	3.76	0.31	14.7%	34.1%	51.2%
Arredondo	Abbott m2000rt RealTime HIV-1	[23]	0.8281	0.13 (−1.00–1.25)			0.35			
Ayele	bioMerieux NucliSens HIV-1 QT	[24]	0.5524	−0.794 (2.90–1.31)	4.64	3.90	0.74	0.0%	31.6%	68.4%
Brambilla	bioMerieux NucliSens HIV-1 QT	[25]	Three patients included, viral loads >3.5 log copies/ml							
Carmona	Abbott m2000rt RealTime HIV-1	[26]	0.5877	−0.218 (−2.95–2.51)	3.76	3.15	0.61	26.6%	27.4%	46.0%
	Roche COBAS Ampliprep/TaqMan v2	[26]	0.7426	0.747 (−1.34–2.83)	3.95	4.08	−0.13	7.5%	43.0%	49.5%
	Roche COBAS Ampliprep/TaqMan v2 FVE		0.7951	−0.096 (−1.81–1.62)	3.25	3.27	−0.02	11.2%	54.6%	34.2%
David	Abbott m2000rt RealTime HIV-1	[27]	0.9409	0.47 (−0.18–1.12)			0.41			
Erba	Abbott m2000rt RealTime HIV-1	[28]	0.7809	0.01 (−0.75–0.77)	4.11	4.03	0.07	0.0%	44.2%	55.8%
Fajardo—capillary	bioMerieux NucliSens EasyQ HIV-1 v2.0	[29]	0.658	−0.35 (−1.26–0.56)						
venous	bioMerieux NucliSens EasyQ HIV-1 v2.0	[29]	0.689	−0.22 (−1.13–0.69)						
Fiscus	bioMerieux NucliSens HIV-1 QT	[30]	0.5988	−0.424 (−3.30–2.46)	4.48	4.20	0.28	22.4%	14.5%	63.2%
Garrido	Abbott m2000rt RealTime HIV-1	[31]	0.8954	0.147 (−0.39–0.68)	3.81	4.15	−0.34	36.1%	43.3%	20.6%
	bioMerieux NucliSens EasyQ HIV-1 v1.1	[31]	0.7585	−0.577 (−2.59–1.43)	3.04	0.00	3.04	36.1%	33.0%	30.9%
Gous	Hologic Aptima		0.7874	−0.134 (−1.30–1.03)	4.04	3.97	0.07	10.0%	40.0%	50.0%
Johannessen	bioMerieux NucliSens EasyQ HIV-1 v1.2	[33]	0.6538	−0.672 (−3.10–1.76)	2.00	0.00	2.00	33.7%	45.9%	20.4%
Lira	bioMerieux NucliSens HIV-1 QT	[36]	0.801	−0.060 (−0.71–0.59)	4.61	4.53	0.07	0.0%	24.6%	75.4%
Lofgren	Abbott m2000rt RealTime HIV-1	[37]	0.9477	−0.083 (−1.26–1.10)	0.00	0.00	0.00	52.7%	15.0%	32.3%
Marconi	Abbott m2000rt RealTime HIV-1	[38]	0.7812	−0.407 (−2.56–1.75)	3.45	3.93	−0.48	15.2%	40.8%	44.0%
Mbida	Abbott m2000rt RealTime HIV-1	[39]	0.8787	−0.272 (−1.87–1.32)	3.62	3.70	−0.08	20.0%	35.6%	44.4%
Mercier-Delarue	bioMerieux NucliSens EasyQ HIV-1 v2.0	[40]	0.7602	−0.544 (−2.54–1.45)	2.77	2.56	0.22	24.5%	42.2%	33.3%
	Roche COBAS Ampliprep/TaqMan v2	[40]	0.5157	0.217 (−2.47–2.91)	2.99	3.44	−0.44	18.8%	40.6%	40.6%
Monleau 2010	Biocentric G2 Generic	[41]		NR			0.45			
Monleau 2013	Abbott m2000rt RealTime HIV-1	[42]	0.4759	0.089 (−3.21–3.39)	0.00	0.00	0.00	59.3%	19.0%	21.7%
	Biocentric G2 Generic	[42]	0.3386	0.926 (−2.38–4.24)	2.58	3.11	−0.53	46.6%	30.5%	22.9%
	bioMerieux NucliSens EasyQ HIV-1 v1.2	[42]	0.8000	−0.476 (−1.89–0.93)	3.76	3.36	0.40	11.0%	48.4%	40.7%
Mwau	Abbott m2000rt RealTime HIV-1		0.6517	−1.169 (−3.44–1.11)	2.19	0.00	2.19	43.2%	37.0%	19.8%
Neogi	Abbott m2000rt RealTime HIV-1	[45]	0.7169	−0.485 (−2.81–1.84)	1.77	0.00	1.77	39.3%	38.7%	22.0%
Onkendi	Roche COBAS Ampliprep/TaqMan v2 FVE	[47]	0.917	−0.200 (−0.63–0.23)						
Ouma	Roche COBAS Amplicor v1.5/TaqMan v2	[48]	0.6575	0.206 (−0.88–1.30)	4.33	4.54	−0.21	1.2%	32.4%	66.4%
Pannus	Roche COBAS Ampliprep/TaqMan v2 FVE	[49]	0.7418	0.296 (−1.06–1.65)	3.54	3.63	−0.01	10.7%	59.1%	39.9%
Pirollo	Siemens VERSANT HIV-1 RNA 1.0 (kPCR)	[50]	0.7697	−0.318 (−2.06–1.42)	3.57	3.25	0.33	13.3%	44.9%	41.8%
Pollack	Roche COBAS Ampliprep/TaqMan v2 SPEX	[51]	0.3701	1.813 (−0.91–4.54)	1.72	3.73	−2.01	34.5%	50.4%	15.1%
	Roche COBAS Ampliprep/TaqMan v2 FVE	[51]	0.6083	0.382 (−1.58–2.34)	3.06	3.30	−0.24	14.2%	64.0%	21.8%
Reigadas	Biocentric	[52]	0.1312	−0.536 (−4.83–3.76)	4.10	3.99	0.11	14.0%	32.6%	53.5%
Rottinghaus	bioMerieux NucliSens EasyQ HIV-1 v1.1	[53]	0.7527	0.054 (−1.36–1.47)	0.00	0.00	0.00	85.0%	7.5%	7.5%
Rutstein—capillary	Abbott m2000rt RealTime HIV-1	[54]	0.81	NR			1.14			
venous	Abbott m2000rt RealTime HIV-1	[54]	0.8649	NR			1.14			
Sahoo	Hologic Aptima	[55]		−0.075 (−0.62–0.48)						
Sawadogo	Roche COBAS Amplicor v1.5/TaqMan v2	[56]	0.3651	1.715 (−1.20–4.63)	1.51	3.76	−2.26	39.9%	44.6%	15.6%
Schmitz	Abbott m2000rt RealTime HIV-1	[57]		0.07–0.09						
Siemens	Siemens VERSANT HIV-1 RNA 1.0 (kPCR)		0.7830	0.002 (−1.24–1.24)	3.63	3.68	−0.05	0.0%	67.4%	32.6%
Taieb	Abbott m2000rt RealTime HIV-1	[58]	0.8432	0.090 (−0.82–0.99)	3.22	4.30	−1.08	58.6%	25.3%	16.2%
	Abbott m2000rt RealTime HIV-1 one-spot	[58]	0.7631	−0.001 (−0.08–0.08)	0.61	0.62	−0.01	34.8%	65.2%	0.0%
	Roche COBAS Ampliprep/TaqMan v2 FVE	[58]	0.6393	−2.090 (−4.44–0.26)	2.78	0	2.78	41.9%	39.9%	18.2%
Tang	Abbott m2000rt RealTime HIV-1 one-spot	[59]	0.9166	0.087 (−0.53–0.71)	4.29	4.02	0.27	65.0%	12.3%	22.7%
Tariro Makadzange	Roche COBAS Ampliprep/TaqMan v2 FVE	[60]	0.782	−1.07 (−0.06–2.16)	5.33	4.26	1.07	58.1%		
Toure Kane	bioMerieux NucliSens EasyQ HIV-1 v1.2	[34]	0.9334	−0.108 (−1.02–0.80)	3.76	3.60	0.16	14.6%	41.5%	43.9%
Uttayamakul	bioMerieux NucliSens HIV-1 QT	[61]	0.667				0.17			
van Deursen	bioMerieux NucliSens EasyQ HIV-1 v2.0	[62]	0.7750	−0.544 (-2.24–1.15)	1.91	0.00	1.91	33.0%	49.5%	17.4%
Vidya	Abbott m2000rt RealTime HIV-1	[63]	0.7069	−0.141 (−1.61–1.32)	4.37	4.39	−0.02	0.0%	39.0%	61.0%
Viljoen	Biocentric	[64]	0.5958	0.277 (−0.64–1.20)	4.33	4.65	−0.32	0.0%	29.7%	70.3%
Waters	Roche COBAS TaqMan RT-PCR	[65]	0.8402	0.209 (−1.33–1.75)	3.24	2.74	0.50	81.3%	9.7%	9.0%
Wu	Roche COBAS Ampliprep/TaqMan v2 FVE	[66]	0.8003	−1.055 (−3.07–0.96)	3.24	0.00	3.24	81.3%	9.7%	9.0%
Yapo	Biocentric	[67]	0.92	0.65 (−1.35–0.06)			0.77			
Yek	Hologic Aptima	[68]	0.7418	0.075 (−1.56–1.71)	3.34	3.49	−0.15	59.6%	26.9%	13.5%
Zeh	Abbott m2000rt RealTime HIV-1	[69]	0.8840	0.264 (−1.33–1.86)	3.68	4.45	−0.77	23.0%	34.5%	42.5%
	Roche COBAS Ampliprep/TaqMan v2	[69]	0.6368	1.002 (−1.80–3.81)	4.01	4.36	−0.35	25.0%	25.0%	50.0%
	Roche COBAS Ampliprep/TaqMan v2 FVE		0.6210	−0.364 (−2.44–1.71)	0.00	0.00	0.00	63.2%	28.1%	8.8%
Zhang	Roche COBAS Ampliprep/TaqMan v2 FVE		0.8615	−0.058 (−1.67–1.56)	1.40	0.00	1.40	48.3%	22.5%	29.2%
Zinyowera	Roche COBAS TaqMan RT-PCR	[43]	0.3080	0.573 (−2.01–3.51)	0.00	0.00	0.00	89.9%	5.5%	4.6%

NR: not reported.

Gray shading: studies included in the meta-analysis.

### Quality of studies

There was some risk of bias in patient selection, however, low risk of bias with the reference standard and index test (S2 Fig). Participants in most studies were not consecutively recruited or failed to report the process of patient recruitment and only 5% of studies reported the process of patient recruitment. There was a high applicability in patient selection, index test, and reference standard; however, there were some concerns as most studies (58%) were carried out in Africa; most studies (>90%) used venous blood prepared in the laboratory with a pipet; and most studies (>90%) used only 1 dried blood spot filter paper (Whatman 903).

### Systematic review analysis

Mean bias was the most commonly reported analytical measurement across all studies included in the systematic review (82%); therefore, forest plots of each study were developed by technology (S3 Fig). Half of the studies included patients on antiretroviral therapy [21–23,29,33,35–37,39,40,42,43,45–47,51–54,56–59,62,64,65,68,69], whereas the remaining studies either included patients not on antiretroviral therapy or did not indicate such information. The study characteristics such as sample size, viral load medians, and patient viral load distributions are summarized in Table 2.

### Meta-analysis

A total of 40 studies provided 45 data sets across the 6 technologies resulting in a total of 10,871 paired dried blood spot and plasma viral load results [22,24,26,28,30,31,33,34,36–45,48–53,56,58,59,62–66,68,69]. Those studies not included from the systematic review were due to primary authors’ inability to sharing data. Of these 58% of pairs were analyzed with the Roche COBAS TaqMan technology [22,26,40,43,48,49,51,56,58,65,66,69], 25% with the Abbott RealTi*m*e HIV-1 technology [26,28,31,37–39,42,45,58,59,63,69], 10% with the bioMérieux NucliSENS EasyQ technology [24,30,31,33,34,36,40,42,53,62], 5% with the Biocentric Generic HIV Charge Virale technology [41,52,64], 1% with the Hologic Aptima [68], and 1% with the Siemens VERSANT HIV-1 RNA technology [50]. Approximately 70% of the paired data points were from studies conducted in Africa [22,24,26,28,33,34,37,39,40,42,43,48,53,56,58,59,64,65,74], of which 36% were from the Southern African Development Community region [22,26,28,42,43,56,59,64] and 24% from the East African Community region [24,33,37,48,65,69].

**Fig 2:**
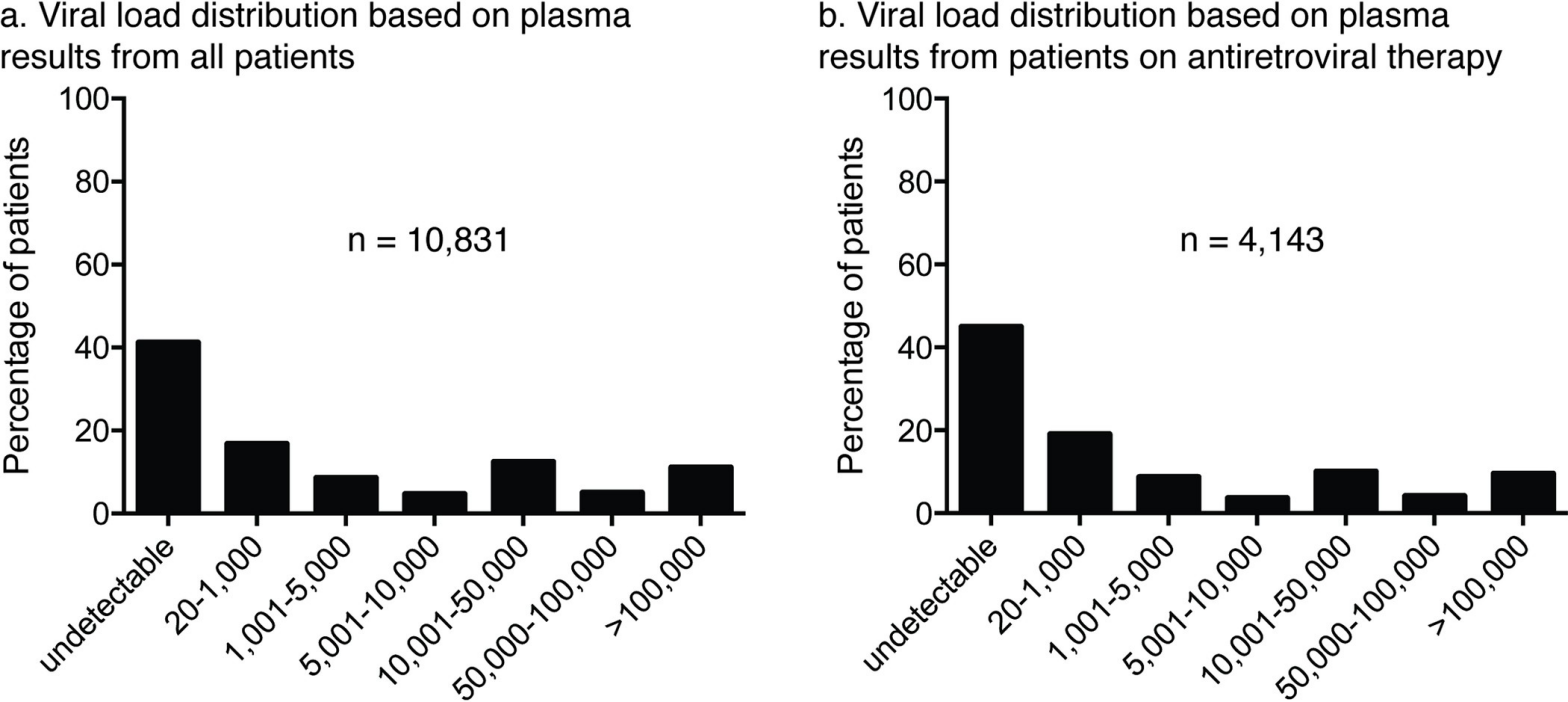
Patient plasma viral load distribution from all studies (a) and including only patients on antiretroviral therapy (b).

The viral load distribution for the 10,871 plasma specimens tested was relatively equally distributed across all viral load ranges (Fig 2A). While approximately 41% of all plasma specimens were undetectable (below the technology’s limit of detection), 30% of all plasma study specimens were between detectable (at or greater than the technology’s limit of detection) and 10,000 copies/ml. Furthermore, when including only plasma specimens from patients known to be on ART, we observed that just over 40% of patients had undetectable levels of viral load (Fig 2B). Approximately 31% of plasma specimens from patients on ART were between detectable and 10,000 copies/ml.

The median dried blood spot viral loads were higher than the median plasma viral loads for all but 2 technologies. Overall, the median difference was 1.03 log copies/ml (Table 3). The Abbott RealTi*m*e HIV-1 two-spot, Abbott RealTi*m*e HIV-1 one-spot, Biocentric Generic HIV Charge Virale, bioMérieux NucliSENS EasyQ HIV-1, Hologic Aptima, Roche COBAS TaqMan FVE, Roche COBAS TaqMan SPEX, and Siemens VERSANT HIV-1 RNA technologies had a difference between the median dried blood spot and plasma specimen viral loads of 0.09, 0.04, 0.17, −0.30, 0.12, 0.33, 1.99, and −0.13 log copies/ml, respectively. The mean bias for each technology was calculated by pooling all primary data for each technology as though one study (Table 3). The mean biases between the dried blood spot and plasma viral load values varied significantly depending on the technology. The overall mean bias was 0.30 log copies/ml. The Abbott RealTi*m*e HIV-1 two-spot (−0.12 log copies/ml), Abbott RealTi*m*e HIV-1 one-spot (0.02 log copies/ml), and Roche COBAS TaqMan FVE (0.06 log copies/ml) assay biases were closest to zero, while the bioMerieux NucliSENS EasyQ HIV-1 (−0.41 log copies/ml) and Roche COBAS TaqMan SPEX (1.03 log copies/ml) assay biases were furthest from zero. The Abbott RealTi*m*e HIV-1 two-spot, bioMerieux NucliSENS EasyQ HIV-1, and Siemens VERSANT HIV-1 RNA technologies had negative mean biases indicating under-quantification compared to the plasma viral load result, which is expected due to the lower input sample volume. The positive mean biases of Biocentric Generic HIV Charge Virale and Roche COBAS TaqMan SPEX reflect over-quantification compared to the plasma viral load result, likely due to processing and extraction chemistries resulting in amplification of total intracellular and extracellular nucleic acids.

**Table 3:** Meta-analysis of clinical metrics overall and for each viral load technology.

		All technologies	Abbott RealTi*m*e HIV-1 two-spot	Abbott RealTi*m*e HIV-1 one-spot	Biocentric Generic HIV Charge Virale	bioMerieux NucliSENS EasyQ HIV-1	Hologic Aptima	Roche COBAS TaqMan HIV-1 FVE	Roche COBAS TaqMan HIV-1 SPEX	Siemens VERSANT HIV-1 RNA
	n	10,831	2,004	700	531	1,062	124	3,076	3,190	144
	**Dried blood spot**									
	Median viral load (log copies/ml)	3.04	3.34	0.68	4.34	2.36	3.65	1.65	3.48	3.46
	**Plasma**									
	Median viral load (log copies/ml)	2.01	3.25	0.64	4.17	2.66	3.53	1.32	1.49	3.59
	Difference in medians (log copies/ml)	1.03	0.09	0.04	0.17	−0.30	0.12	0.33	1.99	−0.13
	Mean bias (log copies/ml)	0.30	−0.12	0.02	0.29	−0.41	0.04	0.06	1.03	−0.22
	**DBS:plasma threshold comparisons**									
**Sensitivity (UCL-LCL)**	1,000:1,000	93.33 (89.85–95.68)	93.13 (83.72–97.27)	88.26 (49.64–98.28)	94.86 (71.14–99.28)	82.95 (78.38–86.71)	84.61 (43.55–97.51)	94.77 (84.59–98.36)	98.23 (95.85–99.26)	90.97 (69.20–97.83)
	3,000:1,000	84.41 (79.07–88.59)	85.41 (69.54–93.75)	83.00 (72.34–90.11)	91.29 (64.11–98.40)	73.93 (64.92–81.29)	71.32 (51.01–85.58)	81.65 (73.29–87.83)	93.32 (88.38–96.25)	74.71 (65.03–82.44)
	5,000:1,000	76.84 (72.36–80.80)	79.13 (60.68–90.31)	81.60 (70.79–89.03)	80.19 (51.31–93.96)	64.54 (55.34–72.78)	62.45 (45.82–76.58)	73.63 (66.69–79.56)	88.32 (81.47–92.85)	68.30 (58.27–76.88)
	7,500:1,000	69.89 (64.96–74.40)	70.30 (52.22–83.68)	76.01 (64.72–84.55)	74.36 (46.21–90.73)	55.96 (46.24–65.25)	54.15 (37.98–69.49)	67.13 (58.14–75.01)	78.66 (69.74–85.49)	62.56 (50.11–73.53)
	10,000:1,000	62.38 (56.42–67.99)	64.52 (46.58–79.13)	71.81 (60.28–81.04)	66.26 (38.27–86.15)	52.76 (42.82–62.48)	51.35 (35.37–67.06)	61.22 (51.90–69.78)	72.15 (63.05–79.74)	54.74 (44.67–64.43)
**Specificity (UCL-LCL)**	1,000:1,000	86.76 (77.83–92.44)	91.11 (82.35–95.75)	99.07 (68.38–99.98)	55.16 (35.01–73.75)	95.06 (89.29–97.80)	73.44 (31.27–94.38)	93.93 (71.95–98.94)	48.49 (22.63–75.18)	87.76 (75.28–94.41)
	3,000:1,000	95.47 (92.18–97.42)	96.29 (92.16–98.28)	99.81 (98.64–99.97)	71.56 (31.04–93.36)	97.16 (91.06–99.14)	90.49 (53.53–98.74)	98.47 (95.47–99.49)	81.68 (54.59–94.29)	97.95 (86.81–99.71)
	5,000:1,000	96.95 (94.60–98.29)	97.15 (94.35–98.59)	99.81 (98.64–99.97)	89.43 (59.20–98.01)	97.45 (92.16–99.20)	94.92 (27.42–99.89)	99.12 (98.02–99.61)	91.37 (75.19–97.37)	97.95 (86.81–99.71)
	7,500:1,000	97.79 (96.17–98.74)	97.80 (95.61–98.91)	99.81 (98.64–99.97)	92.17 (65.70–98.64)	97.57 (93.93–99.05)	96.71 (57.66–99.84)	99.39 (98.58–99.74)	93.14 (84.52–97.13)	97.95 (86.81–99.71)
	10,000:1,000	98.17 (97.08–98.86)	97.96 (95.87–99.00)	99.81 (98.64–99.97)	92.17 (65.70–98.64)	98.18 (94.90–99.37)	96.71 (57.66–99.84)	99.60 (99.18–99.81)	96.92 (90.37–99.06)	97.95 (86.81–99.71)
**Total misclassification (UCL-LCL)**	1,000:1,000	10.69 (7.79–14.50)	8.50 (5.23–13.52)	1.27 (0.04–27.05)	13.71 (3.55–40.67)	9.92 (7.58–12.88)	NS	8.85 (3.85–19.04)	21.16 (11.45–35.78)	9.34 (2.93–26.06)
	3,000:1,000	10.30 (7.65–13.73)	9.27 (5.51–15.17)	1.26 (0.04–26.75)	9.57 (2.46–30.74)	14.05 (9.94–19.51)	NS	7.12 (3.98–12.39)	13.67 (6.05–28.01)	16.89 (11.68–23.81)
	5,000:1,000	12.47 (9.87–15.64)	11.72 (7.32–18.26)	1.31 (0.04–29.26)	14.03 (5.66–30.71)	18.74 (13.10–26.09)	NS	8.94 (5.29–14.71)	11.95 (6.03–22.30)	20.99 (15.16–28.30)
	7,500:1,000	14.97 (12.30–18.10)	14.51 (9.41–21.71)	1.50 (0.04–38.69)	16.74 (8.28–30.94)	22.78 (16.46–30.62)	NS	10.62 (6.26–17.47)	13.25 (8.01–21.14)	25.12 (18.77–32.76)
	10,000:1,000	16.98 (14.13–20.27)	16.99 (11.22–24.91)	1.63 (0.03–45.03)	21.43 (12.95–33.33)	24.79 (18.12–32.93)	NS	12.35 (7.28–20.18)	13.82 (8.93–20.77)	29.74 (22.93–37.59)
**Upward misclassification (UCL-LCL)**	1,000:1,000	14.21 (9.09–21.54)	9.65 (5.00–17.81)	0.93 (0.02–31.62)	44.84 (26.25–64.99)	4.94 (2.20–10.71)	NS	7.63 (1.40–32.52)	51.00 (28.06–73.53)	12.24 (5.59–24.72)
	3,000:1,000	4.78 (2.68–8.38)	4.24 (2.05–8.54)	0.19 (0.03–1.36)	28.44 (6.64–68.96)	2.34 (0.83–6.40)	NS	1.58 (0.45–5.42)	19.63 (7.07–43.97)	2.05 (0.29–13.19)
	5,000:1,000	3.05 (1.71–5.40)	3.39 (1.67–6.78)	0.19 (0.03–1.36)	10.57 (1.99–40.80)	2.34 (0.83–6.40)	NS	0.95 (0.36–2.51)	10.08 (3.67–24.81)	2.05 (0.29–13.19)
	7,500:1,000	2.21 (1.26–3.83)	2.06 (0.87–4.81)	0.19 (0.03–1.36)	7.83 (1.36–34.30)	2.11 (0.73–5.93)	NS	0.61 (0.26–1.42)	6.86 (2.87–15.48)	2.05 (0.29–13.19)
	10,000:1,000	1.77 (1.03–3.02)	1.54 (0.57–4.13)	0.19 (0.03–1.36)	7.83 (1.36–34.30)	1.82 (0.63–5.10)	NS	0.42 (0.20–0.89)	5.06 (2.24–11.01)	2.05 (0.29–13.19)
**Downward misclassification (UCL-LCL)**	1,000:1,000	7.99 (5.75–11.02)	7.42 (3.18–16.39)	11.74 (1.72–50.36)	5.14 (0.72–28.86)	17.05 (13.29–21.62)	NS	6.73 (2.49–16.97)	2.41 (1.09–5.24)	9.03 (2.17–30.80)
	3,000:1,000	16.37 (13.14–20.20)	15.38 (9.20–24.59)	17.00 (9.89–27.66)	8.71 (1.60–35.89)	27.05 (20.54–34.71)	NS	18.67 (12.64–26.71)	7.77 (4.39–13.40)	25.29 (17.56–34.97)
	5,000:1,000	23.16 (19.20–27.64)	21.59 (14.35–31.15)	18.40 (10.97-–29.21)	19.81 (6.04–48.69)	34.87 (26.53–44.26)	NS	26.41 (20.02–33.97)	12.38 (7.65–19.42)	31.70 (23.12–41.73)
	7,500:1,000	30.11 (25.60–35.04)	25.54 (17.72–35.32)	23.99 (15.45–35.28)	25.64 (9.27–53.79)	43.19 (33.84–53.05)	NS	32.87 (24.99–41.86)	21.34 (14.51–30.26)	37.44 (26.47–49.89)
	10,000:1,000	35.94 (31.21–40.97)	31.05 (21.83–42.06)	28.19 (19.00–39.72)	33.74 (13.85–61.73)	47.24 (37.52–57.18)	NS	38.64 (29.55–48.60)	27.75 (20.32–36.65)	45.26 (35.57–55.33)
**PPV (UCL-LCL)**	1,000:1,000	88.20 (82.69–92.13)	91.77 (87.02–94.89)	87.81 (65.23–96.51)	86.46 (43.48–98.15)	94.78 (91.15–96.97)	NS	86.68 (74.19–93.64)	66.02 (44.99–82.19)	92.71 (84.68–96.69)
	3,000:1,000	94.86 (91.34–97.00)	96.18 (92.16–98.18)	93.02 (11.31–99.93)	92.81 (73.64–98.35)	98.22 (96.09–99.20)	NS	95.53 (90.69–97.91)	81.92 (62.50–92.49)	98.54 (90.37–99.80)
	5,000:1,000	96.26 (93.31–97.94)	97.01 (92.97–98.76)	92.97 (11.40–99.93)	96.23 (79.34–99.41)	97.96 (95.53–99.08)	NS	97.38 (94.35–98.80)	88.96 (72.06–96.18)	98.43 (89.66–99.78)
	7,500:1,000	96.93 (94.35–98.35)	97.61 (93.05–99.21)	92.75 (11.80–99.92)	97.88 (82.73–99.78)	97.90 (95.24–99.09)	NS	98.25 (96.44–99.15)	91.74 (76.61–97.41)	98.29 (88.82–99.76)
	10,000:1,000	97.35 (95.08–98.59)	97.95 (92.77–99.44)	92.57 (12.12–99.91)	97.63 (81.07–99.75)	97.89 (95.03–99.12)	NS	98.66 (97.21–99.36)	94.99 (82.96–98.66)	98.01 (87.17–99.72)
**NPV (UCL-LCL)**	1,000:1,000	91.79 (88.17–94.38)	91.11 (81.40–96.00)	99.20 (77.93–99.98)	74.78 (23.33–96.65)	80.93 (69.79–88.62)	NS	96.24 (91.92–98.30)	96.79 (91.90–98.77)	83.30 (59.76–94.37)
	3,000:1,000	85.46 (80.63–89.25)	85.29 (72.82–92.62)	98.86 (53.30–99.98)	69.19 (31.21–91.75)	72.55 (58.77–83.05)	NS	91.14 (83.57–95.42)	92.20 (80.70–97.10)	68.42 (57.18–77.85)
	5,000:1,000	80.91 (75.04–85.67)	80.73 (67.37–89.47)	98.82 (50.27–99.99)	53.99 (18.12–86.15)	66.22 (50.60–78.96)	NS	88.23 (78.92–93.75)	89.19 (73.55–96.07)	63.41 (52.51–73.09)
	7,500:1,000	76.31 (69.79–81.79)	75.81 (62.41–85.54)	98.66 (40.31–99.99)	48.35 (13.64–84.72)	62.08 (46.78–75.30)	NS	85.94 (75.22–92.49)	83.43 (63.97–93.46)	59.08 (48.55–68.83)
	10,000:1,000	73.29 (66.31–79.27)	72.52 (58.99–82.88)	98.55 (34.63–99.99)	41.72 (10.32–81.66)	60.29 (45.17–73.68)	NS	83.96 (72.18–91.35)	80.38 (58.61–92.22)	54.73 (44.66–64.43)

NS: insufficient data to perform meta-analysis.

**Fig 3:**
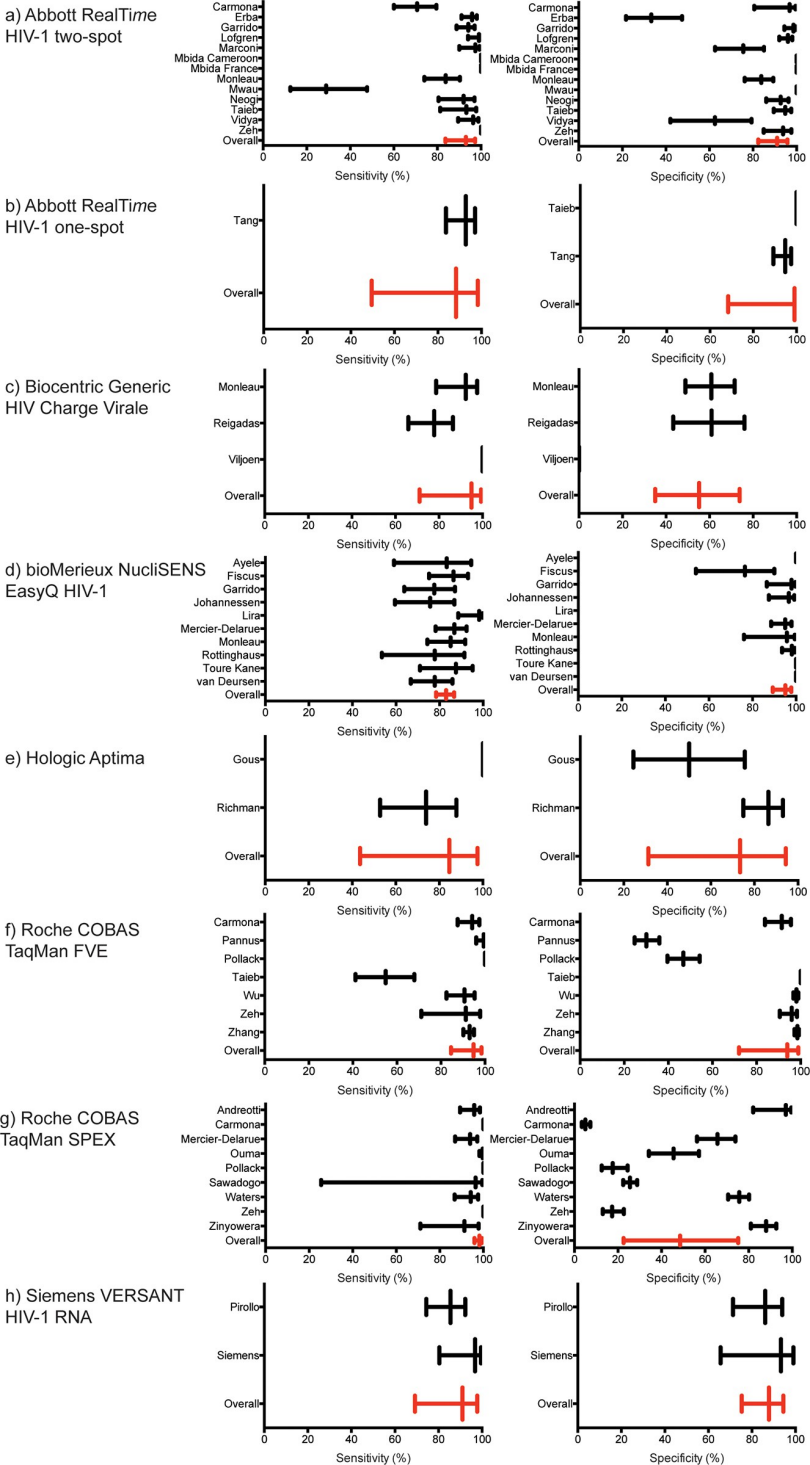
Forest plots of sensitivity and specificity of all studies included in the meta-analysis for each viral load technology using a treatment failure threshold of 1,000 copies/ml. Abbott RealTi*m*e HIV-1 two-spot (a), Abbott RealTi*m*e HIV-1 one-spot (b), Biocentric Generic HIV Charge Virale (c), bioMerieux NucliSENS EasyQ HIV-1 (d), Hologic Aptima (e), Roche COBAS TaqMan FVE (f), Roche COBAS TaqMan SPEX (g), Siemens VERSANT HIV-1 RNA (h). Red bars and lines indicate the overall metrics for each viral load technology.

WHO and many national clinical guidelines in resource-limited settings recommend using viral load testing as a binary result, above or below a specific threshold to identify treatment failure. We, therefore, compared several treatment failure thresholds for dried blood spot specimens (1,000, 3,000, 5,000, 7,500, and 10,000 copies/ml) to the currently suggested 1,000 copies/ml threshold for plasma specimens for correctly classifying patients (Table 3 and Fig 3). Using a dried blood spot specimen threshold of 1,000 copies/ml, all 6 technologies had a sensitivity of detecting a viral load above 1,000 copies/ml of greater than 80%. At the same threshold, the specificity of detecting a viral load below 1,000 copies/ml was over 80% for all technologies except for the Biocentric Generic HIV Charge Virale (55.16%), Hologic Aptima (73.44%), and Roche COBAS TaqMan SPEX (43.86%). Using a higher treatment failure threshold, such as 5,000 copies/ml, for dried blood spot specimens reduced the sensitivity and increased the specificity of all technologies. Finally, HSROC curves were created for those technologies where more than 4 studies were included in the meta-analysis (S4 Fig).

Additionally, to better understand the performance of dried blood spot specimens at lower treatment failure thresholds (below 1,000 copies/ml), we compared the 6 predefined treatment treatment failure thresholds—detectable, 200, 400, 500, 600, and 800 copies/ml—between dried blood spot specimens and plasma for each technology and protocol (Table 4). The Biocentric Generic HIV Charge Virale and Roche COBAS TaqMan SPEX technologies had poor specificity (<40%) at all lower thresholds below 1,000 copies/ml. The Siemens Versant had a sensitivity and specificity above 85% when using a threshold of 800 copies/ml; however, the specificity declined to below 80% at a threshold of 600 copies/ml and below 70% with all thresholds below 500 copies/ml. The Abbott RealTi*m*e HIV-1 two-spot and Roche COBAS TaqMan FVE protocols had high sensitivities and specificities at all lower thresholds except detectable. The new Abbott RealTi*m*e HIV-1 one-spot protocol, however, had high specificities at all lower thresholds, but sensitivity performance was below 85% at the 200 copies/ml and detectable thresholds. The Hologic Aptima had high sensitivities with all thresholds except detectable; however, specificity was lower than 85% at the thresholds of 800 copies/ml and 200 copies/ml. Finally, the bioMérieux NucliSENS EasyQ HIV-1 had sensitivities and specificities greater than 85% at all thresholds.

**Table 4:** Meta-analysis of clinical metrics overall and for each viral load technology and protocol for treatment failure thresholds below 1,000 copies/ml.

		All technologies	Abbott RealTi*m*e HIV-1 two-spot	Abbott RealTi*m*e HIV-1 one-spot	Biocentric Generic HIV Charge Virale	bioMerieux NucliSENS EasyQ HIV-1	Hologic Aptima	Roche COBAS TaqMan HIV-1 FVE	Roche COBAS TaqMan HIV-1 SPEX	Siemens VERSANT HIV-1 RNA
	n	10,831	2,004	700	531	1,062	124	3,076	3,190	144
	**DBS:plasma threshold comparisons**									
**Sensitivity (UCL-LCL)**	800:800	95.04 (91.45–97.17)	92.59 (82.86–96.99)	91.55 (4.60–99.96)	98.64 (43.94–99.99)	85.36 (80.27–89.32)	93.47 (31.10–99.78)	95.35 (87.11–98.42)	99.70 (95.62–99.98)	91.07 (74.87–97.22)
	600:600	95.24 (92.21–97.12)	92.71 (84.14–96.83)	92.95 (0.04–100.00)	98.55 (60.16–99.97)	88.87 (83.99–92.40)	94.54 (27.75–99.87)	94.17 (83.85–98.05)	99.26 (96.03–99.87)	93.45 (84.10–97.47)
	500:500	95.43 (92.38–97.30)	93.11 (84.49–97.11)	92.96 (0.00–100.00)	98.40 (66.61–99.95)	89.04 (84.76–92.22)	94.50 (29.45–99.86)	93.37 (81.99–97.75)	99.22 (95.81–99.86)	97.21 (66.06–99.84)
	400:400	95.51 (92.35–97.41)	92.48 (84.11–96.61)	94.36 (0.00–100.00)	97.79 (60.28–99.92)	90.17 (85.52–93.44)	94.69 (28.03–99.88)	92.26 (80.79–97.13)	99.36 (95.26–99.92)	97.18 (62.91–99.86)
	200:200	94.78 (91.11–96.99)	90.80 (82.55–95.37)	97.18 (0.00–100.00)	98.09 (65.21–99.93)	89.42 (83.74–93.28)	95.01 (22.04–99.92)	89.86 (76.26–96.07)	99.16 (94.75–99.87)	97.67 (71.68–99.86)
	Detectable	95.39 (90.12–97.91)	92.81 (76.47–98.09)	93.13 (62.76–99.09)	97.98 (59.94–99.94)	88.59 (75.29–95.18)	75.42 (51.82–89.75)	97.10 (58.02–99.88)	99.76 (94.64–99.99)	90.08 (83.66–94.15)
**Specificity (UCL-LCL)**	800:800	83.56 (71.57–91.12)	92.01 (83.14–96.41)	99.77 (23.66–100.00)	38.14 (10.78–75.88)	95.99 (91.31–98.20)	72.16 (41.80–90.34)	92.86 (64.86–98.92)	37.59 (13.30–70.28)	86.62 (68.28–95.11)
	600:600	82.52 (69.82–90.60)	92.54 (81.42–97.23)	99.77 (12.38–100.00)	28.13 (5.97–70.70)	95.21 (91.29–97.42)	89.34 (50.48–98.57)	92.95 (68.03–98.79)	32.98 (11.54–64.98)	78.77 (60.95–89.82)
	500:500	80.41 (66.80–89.33)	93.16 (81.96–97.61)	99.77 (9.33–100.00)	23.72 (4.28–68.41)	95.27 (91.10–97.54)	89.06 (50.38–98.49)	91.71 (67.71–98.32)	29.63 (9.78–62.04)	65.63 (30.99–89.04)
	400:400	79.81 (65.43–89.20)	93.15 (80.02–97.88)	99.77 (8.20–100.00)	11.35 (0.61–72.77)	95.61 (90.73–97.98)	88.44 (47.82–98.46)	92.04 (67.80–98.45)	27.71 (9.04–59.65)	64.91 (24.64–91.28)
	200:200	81.57 (67.54–90.40)	97.22 (91.66–99.11)	99.78 (5.34–100.00)	15.09 (1.30–70.49)	92.94 (89.26–95.43)	81.48 (71.52–88.52)	91.60 (71.21–97.96)	25.31 (7.60–58.26)	64.68 (26.23–90.41)
	Detectable	60.98 (34.29–82.40)	78.79 (8.46–99.33)	93.16 (66.40–98.94)	18.62 (4.87–50.58)	93.46 (90.43–95.59)	87.18 (66.58–95.87)	58.09 (6.37–96.58)	4.25 (0.17–53.54)	69.23 (40.93–87.96)
**Total misclassification (UCL-LCL)**	800:800	9.97 (6.92–14.15)	8.39 (5.26–13.11)	0.23 (0.00–78.78)	11.96 (1.98–47.79)	9.03 (6.80–11.91)	18.55 (12.65–26.37)	10.35 (5.77–17.89)	22.44 (11.82–38.44)	10.39 (4.12–23.82)
	600:600	9.12 (6.16–13.30)	8.04 (5.01–12.67)	0.23 (0.00–84.65)	12.85 (1.95–52.18)	7.78 (5.64–10.64)	6.67 (0.54–48.42)	10.42 (6.16–17.09)	23.81 (12.52–40.57)	11.11 (6.92–17.37)
	500:500	9.21 (6.15–13.58)	7.72 (4.78–12.24)	0.23 (0.00–87.30)	10.79 (1.09–57.09)	7.82 (5.72–10.61)	6.67 (0.54–48.43)	11.03 (7.08–16.79)	25.00 (13.21–42.19)	13.19 (8.58–19.76)
	400:400	8.87 (5.80–13.35)	7.94 (5.04–12.29)	0.23 (0.00–87.36)	9.40 (0.62–63.44)	7.50 (5.25–10.62)	6.55 (0.49–50.14)	10.95 (7.18–16.33)	25.35 (13.21–43.11)	13.19 (8.58–19.76)
	200:200	8.04 (4.99–12.70)	7.13 (4.11–12.08)	0.23 (0.00–88.53)	4.33 (0.08–73.07)	8.94 (6.41–12.34)	6.23 (0.25–63.87)	11.01 (7.52–15.83)	24.02 (12.02–42.26)	10.42 (6.38–16.56)
	Detectable	8.73 (5.22–14.24)	9.64 (4.17–20.74)	15.00 (12.54–17.84)	4.65 (0.07–76.18)	11.26 (6.16–19.71)	20.97 (14.69–29.02)	7.29 (1.41–30.10)	8.26 (2.36–25.14)	11.81 (7.47–18.17)
**Upward misclassification (UCL-LCL)**	800:800	16.44 (8.88–28.43)	7.99 (3.59–16.86)	0.23 (0.00–76.34)	61.86 (24.12–89.22)	4.01 (1.80–8.69)	27.84 (9.66–58.20)	7.14 (1.08–35.14)	62.41 (29.72–86.70)	13.38 (4.89–31.72)
	600:600	17.48 (9.40–30.18)	7.46 (2.77–18.58)	0.23 (0.00–87.62)	71.87 (29.30–94.03)	4.79 (2.58–8.71)	10.66 (1.43–49.52)	7.05 (1.21–31.97)	67.02 (35.02–88.46)	21.23 (10.18–39.05)
	500:500	19.59 (10.67–33.20)	6.84 (2.39–18.04)	0.23 (0.00–90.67)	76.28 (31.59–95.72)	4.73 (2.46–8.90)	10.94 (1.51–49.62)	8.29 (1.68–32.29)	70.37 (37.96–90.22)	34.37 (10.96–69.01)
	400:400	20.19 (10.80–34.57)	6.85 (2.12–19.98)	0.23 (0.00–91.80)	88.65 (27.23–99.39)	4.39 (2.02–9.27)	11.56 (1.54–52.18)	7.96 (1.55–32.20)	72.29 (40.35–90.96)	35.09 (8.72–75.36)
	200:200	18.43 (9.60–32.46)	2.78 (0.89–8.34)	0.22 (0.00–94.66)	84.91 (29.51–98.70)	7.06 (4.57–10.74)	18.52 (11.48–28.48)	8.40 (2.04–28.79)	74.69 (41.74–92.40)	35.32 (9.59–73.77)
	Detectable	39.02 (17.60–65.71)	21.21 (0.67–91.54)	6.84 (1.06–33.60)	81.38 (49.42–95.13)	6.54 (4.41–9.57)	12.82 (4.13–33.42)	41.91 (3.42–93.63)	95.75 (46.46–99.83)	30.77 (12.04–59.07)
**Downward misclassification (UCL-LCL)**	800:800	4.96 (2.83–8.55)	7.41 (3.01–17.14)	8.45 (0.04–95.40)	1.36 (0.01–56.06)	14.64 (10.68–19.73)	6.53 (0.22–68.90)	4.65 (1.58–12.89)	0.30 (0.02–4.38)	8.93 (2.78–25.13)
	600:600	4.76 (2.88–7.79)	7.29 (3.17–15.86)	7.05 (0.00–99.96)	1.45 (0.03–39.84)	11.13 (7.60–16.01)	5.46 (0.13–72.25)	5.83 (1.95–16.15)	0.74 (0.13–3.97)	6.55 (2.53–15.90)
	500:500	4.57 (2.70–7.62)	6.89 (2.89–15.51)	7.04 (0.00–100.00)	1.60 (0.05–33.39)	10.96 (7.78–15.24)	5.50 (0.14–70.55)	6.63 (2.25–18.01)	0.78 (0.14–4.19)	2.79 (0.16–33.94)
	400:400	4.49 (2.59–7.65)	7.52 (3.39–15.89)	5.64 (0.00–100.00)	2.21 (0.08–39.72)	9.83 (6.56–14.48)	5.31 (0.12–71.97)	7.74 (2.87–19.21)	0.64 (0.08–4.74)	2.82 (0.14–37.09)
	200:200	5.22 (3.01–8.89)	9.20 (4.63–17.45)	2.82 (0.00–100.00)	1.91 (0.07–34.79)	10.58 (6.72–16.26)	4.99 (0.08–77.96)	10.14 (3.93–23.74)	0.84 (0.13–5.25)	2.33 (0.14–28.32)
	Detectable	4.61 (2.09–9.88)	7.19 (1.91–23.53)	6.87 (0.91–37.24)	2.02 (0.06–40.06)	11.41 (4.82–24.71)	24.58 (10.25–48.18)	2.90 (0.12–41.98)	0.24 (0.01–5.36)	9.92 (5.85–16.34)
**PPV (UCL-LCL)**	800:800	89.22 (82.88–93.40)	93.43 (88.98–96.16)	86.67 (11.76–99.69)	88.79 (50.70–98.39)	96.91 (93.16–98.64)	63.04 (48.39–75.64)	87.09 (66.28–95.86)	67.49 (40.14–86.53)	93.44 (82.58–97.71)
	600:600	89.74 (83.48–93.80)	94.46 (89.47–97.16)	82.50 (13.38–99.31)	88.64 (48.24–98.49)	96.13 (91.82–98.21)	93.68 (8.52–99.96)	89.28 (68.33–96.98)	66.39 (38.39–86.23)	91.18 (83.90–95.35)
	500:500	89.50 (83.10–93.66)	95.13 (89.88–97.73)	80.49 (31.57–97.36)	90.95 (47.06–99.13)	96.08 (91.90–98.14)	93.66 (9.34–99.95)	87.39 (69.63–95.44)	66.21 (37.81–86.33)	88.75 (78.93–94.33)
	400:400	90.26 (83.87–94.29)	95.37 (90.57–97.79)	79.76 (21.07–98.31)	92.82 (45.93–99.49)	96.25 (96.23–96.26)	93.67 (8.17–99.96)	86.58 (72.16–94.13)	66.90 (37.97–86.96)	89.88 (78.90–95.47)
	200:200	93.09 (86.54–96.58)	99.14 (93.84–99.89)	77.53 (66.60–85.65)	96.79 (38.29–99.93)	95.31 (90.04–97.86)	93.81 (7.53–99.96)	87.80 (75.05–94.51)	70.56 (39.94–89.62)	92.17 (85.64–95.88)
	Detectable	97.60 (94.45–98.98)	99.69 (91.78–99.99)	93.25 (53.87–99.39)	96.80 (38.43–99.93)	96.51 (93.13–98.26)	93.69 (35.94–99.75)	98.07 (90.92–99.61)	92.19 (70.22–98.34)	98.67 (65.40–99.97)
**NPV (UCL-LCL)**	800:800	92.83 (87.27–96.07)	90.48 (79.65–95.85)	99.77 (48.10–100.00)	33.65 (1.43–94.65)	79.41 (62.31–90.00)	92.31 (83.92–96.50)	97.39 (94.33–98.82)	97.53 (94.81–98.84)	78.00 (64.49–87.38)
	600:600	92.44 (87.13–95.67)	89.59 (77.67–95.52)	99.77 (56.44–100.00)	41.43 (3.28–93.66)	83.11 (64.24–93.09)	97.05 (31.73–99.96)	96.66 (92.95–98.45)	96.85 (93.97–98.38)	83.33 (68.95–91.84)
	500:500	92.27 (86.93–95.55)	89.73 (79.34–95.21)	99.77 (55.62–100.00)	34.65 (2.09–92.93)	83.19 (65.11–92.92)	97.00 (32.11–99.95)	95.92 (91.92–97.98)	96.20 (92.60–98.08)	83.39 (53.74–95.60)
	400:400	91.83 (85.88–95.41)	88.15 (75.52–94.72)	99.77 (67.05–100.00)	26.51 (2.48–83.67)	84.74 (70.14–92.92)	93.24 (84.77–97.16)	94.71 (89.03–97.53)	95.21 (90.77–97.57)	81.23 (49.15–95.09)
	200:200	87.86 (80.59–92.66)	82.53 (71.11–90.07)	99.79 (91.24–100.00)	14.76 (0.24–92.55)	79.75 (63.92–89.74)	96.57 (27.65–99.95)	92.24 (84.69–96.23)	93.38 (85.67–97.08)	79.31 (60.95–90.40)
	Detectable	57.74 (41.04–72.84)	58.92 (35.49–78.90)	93.33 (50.20–99.49)	3.19 (0.01–95.43)	63.22 (33.93–85.19)	68.34 (38.97–87.94)	52.90 (27.86–76.57)	72.19 (36.12–92.26)	21.12 (0.86–89.25)

**Fig 4:**
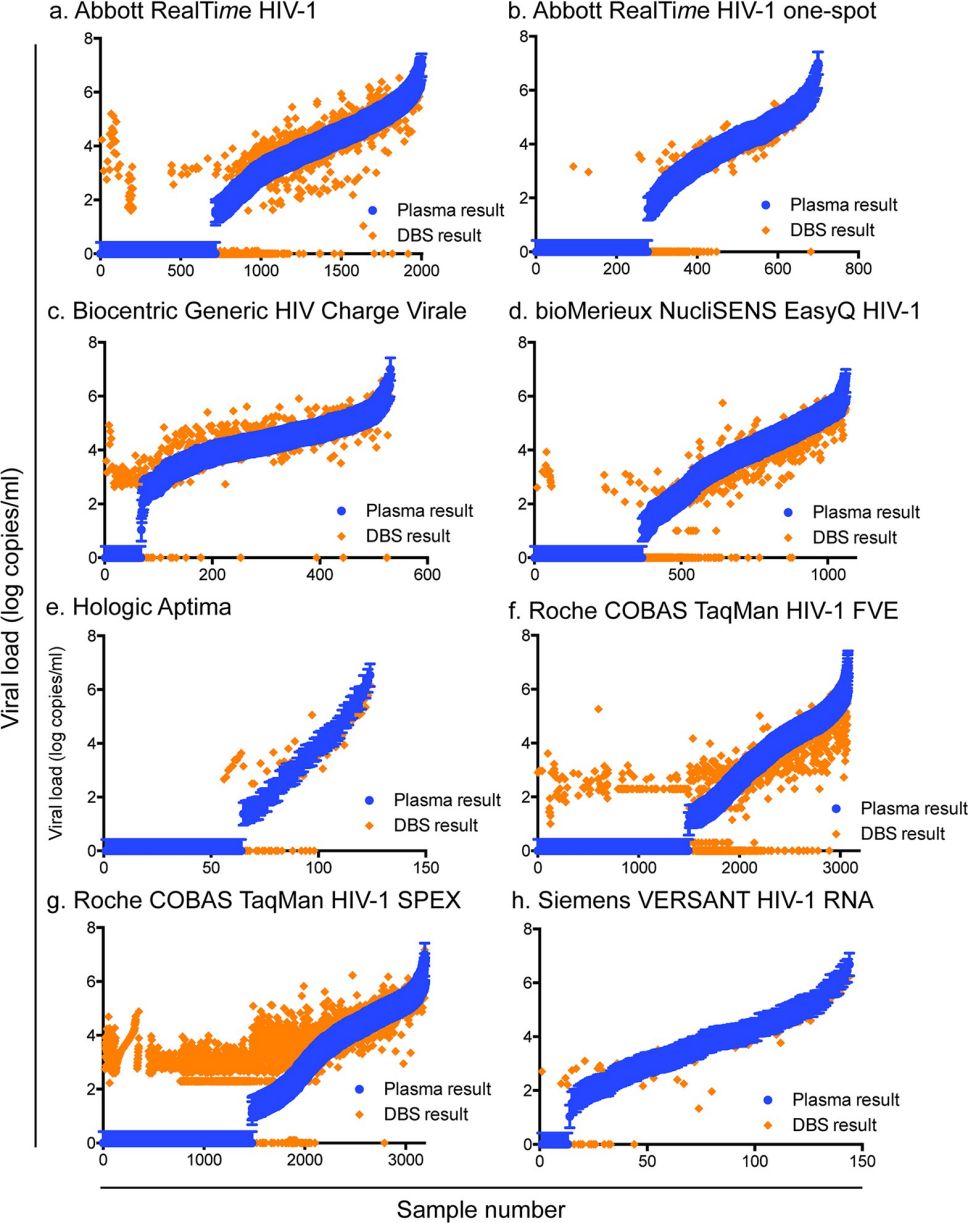
A substantial proportion of dried blood spot results fall outside of the plasma result +/−0.3 log copies/ml for each technology. Abbott RealTi*m*e HIV-1 two-spot (a), Abbott RealTi*m*e HIV-1 one-spot (b), Biocentric Generic HIV Charge Virale (c), bioMerieux NucliSENS EasyQ HIV-1 (d), Hologic Aptima (e), Roche COBAS TaqMan FVE (f), Roche COBAS TaqMan SPEX (g), Siemens VERSANT HIV-1 RNA (h). Blue bars represent +/−0.3 log copies/ml of the plasma result, while orange triangles represent the paired dried blood spot viral load result.

Quantitative polymerase chain reaction inherently introduces a level of variability in test results, generally +/−0.3 log copies/ml [68,69]. We, therefore, sought to understand if the performance observed with each technology was within the inherent assay variability limits. For the Abbott RealTi*m*e HIV-1 two-spot, Abbott RealTi*m*e HIV-1 one-spot, Biocentric Generic HIV Charge Virale, bioMérieux NucliSENS EasyQ HIV-1, Hologic Aptima, Roche COBAS TaqMan FVE, Roche COBAS TaqMan SPEX, and Siemens VERSANT HIV-1 RNA technologies, 59.28%, 68.71%, 38.04%, 52.54%, 50.40%, 62.03%, 33.45%, 47.22% of dried blood spot specimen test results were within the standard deviation of +/−0.3 log copies/ml of the paired plasma test result, respectively (Fig 4).

### Sensitivity analyses

**Fig 5:**
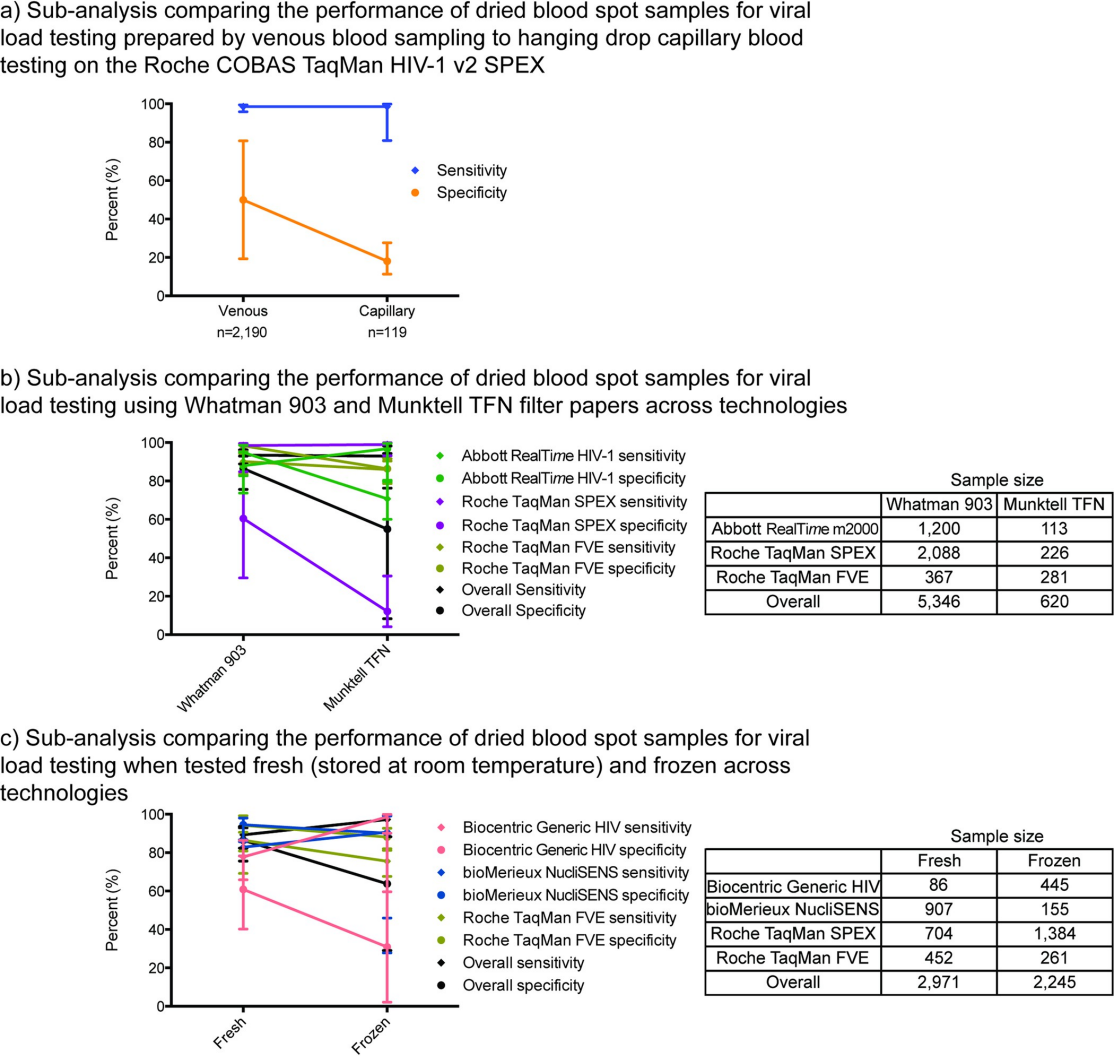
Meta-analysis sub-analyses. Sensitivity and specificity sub-analyses by venous and capillary preparation (a), dried blood spot filter card type (b), and storage temperature (c) compared to plasma with a treatment failure threshold of 1,000 copies/ml.

We next conducted sensitivity analyses with subsets of the data specific to each technology to understand possible causes of lower analytical and clinical performance (Figs 5 and S5). After including only studies in which manufacturer-recommended procedures for dried blood spot specimen collection and processing were precisely followed, we observed no change in the performance of dried blood spot specimens on each of the viral load platforms (S5 Fig). Similarly, performance of dried blood spot specimens did not change when used with older and newer versions of the assays as well as within ART and ART-naïve patient populations (S5 Fig).

Dried blood spot specimens can be prepared and stored in various ways. We, therefore, performed sub-analyses to detect differences in dried blood spot specimen test performance due to venous or capillary blood specimen collection, dried blood spot filter card type, and storage temperature. While the sample size for the capillary specimen collection sub-analysis was relatively small (*n* = 119), the specificity was reduced compared with venous blood specimens (Fig 5A). No significant difference was observed between the performance of the Munktell TFN filter card and Whatman 903 card types on each of the viral load technologies analyzed (Fig 5B). Similarly, no significant difference was found between the performance of dried blood spot specimens kept at room temperature or frozen (Fig 5C).

## Discussion

Dried blood spot specimens may increase access to HIV viral load testing for virological monitoring of ART patients in resource-limited settings. This study analyzed the technical performance of laboratory-based viral load technologies for accurate quantification of viral load using dried blood spot specimens compared to plasma specimens. While the performance varied between technologies, the most commonly used viral load technologies, Abbott RealTi*m*e HIV-1, bioMérieux NucliSENS EasyQ HIV-1, Roche COBAS TaqMan using the FVE protocol, and Siemens VERSANT HIV-1 RNA performed best and within acceptable limits when using a treatment failure threshold of 1,000 copies/ml. Further, utilizing the same treatment failure threshold for both plasma and dried blood spot specimens will avoid differentiated training and interpretation by specimen type and facilitate viral load scale-up in resource-limited settings. Additionally, this analysis indicated that the use of higher thresholds (e.g., 3,000 or 5,000 copies/ml) for dried blood spot specimen viral load testing will result in higher levels of misclassification of treatment failure with most viral load testing technologies.

The sensitivity for 5 technologies were above 90%, however, between 80% to 90% for the Abbott RealTi*m*e HIV-1 one-spot, bioMérieux NucliSENS EasyQ HIV-1, and Hologic Aptima assays using a treatment failure threshold of 1,000 copies/ml. Increasing the treatment failure threshold further reduces the sensitivities of all assays. This performance indicated that most patients with an elevated viral load would be identified by all technologies; however, it is important to note that some patients failing treatment will be missed and only retested a year later when using technologies with lower sensitivity. Additionally, 5 technologies had a specificity above 85% using the treatment failure threshold of 1,000 copies/ml. Two technologies, Biocentric Generic HIV Charge Virale and Roche COBAS TaqMan using the SPEX protocol had low specificities of approximately 55% and 44%, respectively, using a treatment failure threshold of 1,000 copies/ml. This performance would be concerning as significant proportions of patients suppressing their viral load would be incorrectly identified as failing treatment; however, additional viral load measurements are required prior treatment switching and thus this misclassification could be resolved. Though some variability was observed with the Roche COBAS TaqMan using the FVE protocol in the meta-analysis, recent studies have shown consistent performance with sensitivity and specificity greater than 90% [51,60]. Furthermore, the Hologic Aptima had a sensitivity of approximately 75%; however, it is important to note that this is a relatively new technology and only 2 studies were included in the meta-analysis. Additional data may support more precise estimates.

Furthermore, though technologies have variable performance when using dried blood spot specimens at lower treatment failure thresholds, several technologies and protocols can be considered if lower treatment failure thresholds are required. While the lower limits of detection of dried blood spot specimens tend to be higher than plasma due to the smaller specimen volume that clearly does not preclude the technologies or protocols from correctly classifying patients as failing or not failing treatment, the most critical clinical measurement used. Therefore, limits of detection should not be the primary metric for consideration when reviewing technology performance or selecting one for routine use. Prior to changing guidelines and putting in the significant work to implement such an algorithm change, it is important to consider a range of factors, including the prevalence and clinical relevance of low-level viremia, especially in relation to implementation and scale-up of optimized antiretroviral drugs such as dolutegravir, access to testing, result utilization, and other scale-up priorities to ensure that patients have access to high-quality testing and necessary follow-up clinical care.

The sensitivity analyses performed in this study suggest that low adherence to manufacturer-recommended dried blood spot specimen collection and extraction protocols, study geographies, and technology version used did not contribute significantly to any observed differences in dried blood spot and plasma specimen test results. Though the sensitivity analysis including only data generated through compliance with the manufacturers’ recommended protocols did not improve the performance of dried blood spot specimens for viral load testing, manufacturer protocols should be precisely followed for optimal technology performance. Performance variability between test platforms is likely due to different dried blood spot nucleic acid extraction methods and viral load test chemistries employed by each platform. This was observed with the standard Roche COBAS TaqMan SPEX and FVE specimen extraction protocols; the latter protocol was associated with much higher levels of specificity when compared with paired plasma specimen testing. Similarly, the lower specificity of the Biocentric Generic HIV Charge Virale test used in the studies analyzed may reflect a need to optimize specimen extraction for dried blood spot specimens.

Viral load testing is known to be inherently variable within limits [76,77]; however, 50% to 60% of the dried blood spot specimen test results on all platforms fell outside the inherent variability commonly accepted to exist in viral load tests, +/−0.3 log copies/ml, of the plasma specimen test result. This highlights the effect of dried blood spot use on variability. The clinical relevance and interpretation of this additional variability is, however, unclear given that a binary threshold above or below 1,000 copies/ml is recommended by WHO to define treatment failure. Further refinement of dried blood spot protocols and testing may allow for more careful quantitative use of this specimen type in the future.

This systematic review and meta-analysis had some limitations. Almost all studies in this systematic review and meta-analysis used dried blood spots prepared in laboratories using precision pipetting for consistent measurement of input specimen volumes. Hence, the findings may not reflect performance in the field with less precise specimen collection procedures such as allowing the blood drops to drip from the patient’s finger or heel directly onto filter cards as practiced for HIV early infant diagnosis dried blood spot specimen collection [29,43,54,57,59]. Further analysis of the threshold in the context of the finger-prick/“hang drop” blood sample collection method would be beneficial. Incompletely filled circles, as potentially could be observed in the field using the hanging drop technique, may result in inaccurate test results due to variable blood volumes. Viral load testing, however, is quantitative and requires a precise known amount of input volume, though the impact of variability in input volume, and sampling approaches such as the “hang drop” technique, on clinical misclassification needs further study before use. Accordingly, WHO has recommended the use of precision pipets or measured micro-capillary EDTA tubes to ensure the appropriate volume is added to each specimen spot [78]. Additionally, sample storage and transportation are critically important for dried blood spot specimens; however, this systematic review was unable to review the effect of inappropriate storage and transportation on clinical performance. Furthermore, almost all studies in this systematic review and meta-analysis used the Whatman 903 dried blood spot paper; therefore, additional studies may be needed to better validate other types of dried blood spot collection papers for use with viral load testing. Finally, though nearly 70% of studies provided primary data for inclusion in the meta-analysis, the possibility exists that the data and results of those not included could be slightly different or that the current data set may be biased; however, no clear differences were observed between those studies that provided primary data and those that were unable to do so.

The sample sizes for each sub-analysis were small; therefore, further studies are warranted to better understand the performance of dried blood spot specimens under these conditions. Abbott has recently changed their dried blood spot protocol to only use 1 dried blood spot and a different dried blood spot extraction buffer. Data on the new protocol are very limited; however, 2 studies reviewed the performance and found sensitivity and specificity of greater than 90% [58,59]. While additional critical analytical parameters are important such as analytical performance and sensitivity, we focused on the clinical implications. As with all systematic reviews, the search strategy may have missed some titles; however, a significant number of studies and data points were included in both the systematic review and meta-analysis, thus limiting potential biases. Additionally, the time of sample storage was rarely included or defined. Finally, no studies reviewed the use of dried blood spot samples for viral load testing within the longitudinal algorithm currently recommended by the WHO [79].

While this systematic review and meta-analysis informs the use of dried blood spot specimens for viral load testing [79], it is important to note that the performance characteristics described herein used single cross-sectional specimens. WHO guidelines, however, defines treatment failure as 2 consecutive viral load tests greater than 1,000 copies/ml that are at least 3 months apart with adherence counseling after the first viral load. The technical performance of dried blood spot specimens within such a longitudinal treatment failure algorithm is unknown and should be studied further. Furthermore, few manufacturers have sought regulatory approval to use dried blood spot samples for viral load testing. Currently, only the Abbott RealTi*m*e HIV-1 one-spot and bioMérieux NucliSENS EasyQ HIV-1 have CE-IVD approval and/or WHO prequalification listing. It is critical, however, for manufacturers to seek regulatory approval to provide better support to countries in need of this additional sample type to further expand access.

This study raises an important issue of understanding appropriate and acceptable levels of clinical misclassification for current treatment failure thresholds. The acceptable level of misclassification with viral load tests is, however, unclear and further investigation may be warranted to guide technology development and selection.

The gold standard biomarker for viral load testing is circulating HIV RNA in plasma [8,9,11,13,80]. Because plasma viral load testing is not accessible to many HIV patients in resource-limited settings, countries and companies have sought alternative specimen types. Whole blood specimens, however, result in elevated viral loads compared to plasma specimens because of the detection of intracellular RNA and DNA. Alternative specimen input types, such as whole blood, has sparked debate as to the most appropriate biomarker(s) for detecting treatment failure [77,78]. Additional research would provide better insight as to the clinical and virological relevance of intracellular nucleic acids in detecting treatment failure.

The meta-analysis presented here offers advantages over previous systematic reviews [81–83], which were limited in robustness by variations in the analysis methodology and sample sizes of individual studies. Systematic reviews also do not allow for data manipulation or sensitivity analyses to draw new relevant conclusions. Meta-analyses have more power to detect real differences compared to individual studies or systematic reviews given that precision estimates increase with pooled data. Further, meta-analyses provide greater insights into the consistency of test results through stronger quantification of the variability of test accuracy across many settings and technologies.

In conclusion, though variability was seen between technologies included, several technologies performed best using a treatment failure threshold of 1,000 copies/ml. The Abbott RealTi*m*e HIV-1 two- and one-spot, bioMérieux NucliSENS EasyQ HIV-1, Roche COBAS TaqMan using the FVE protocol, and Siemens VERSANT HIV-1 RNA technologies can all be considered for use with dried blood spot specimens when prepared using precision pipets or measured micro-capillary EDTA tubes with a treatment failure threshold of 1,000 copies/ml. It is expected that increased interest in and implementation of viral load testing using dried blood spot specimens may encourage manufacturers to acquire regulatory approval for this specimen type for in vitro diagnostic use, while new technologies such as plasma separation cards may further support the testing landscape.

## Supporting information

S1 PRISMA Checklist PRISMA 2020 Checklist. (PDF)

S1 Fig Flow diagram of updated full search. (TIF)

S2 Fig QUADAS-2 scores of included studies. (TIF)

S3 Fig Forest plots of mean bias and limits of agreement for each study included in the systematic review for each technology. (TIF)

S4 Fig HSROC curves of each study included in the meta-analysis by technology with a treatment failure threshold of 1,000 copies/mL. Circle sizes were relative to the study sample size. The red square was the pooled summary point.
(TIF)

S5 Fig Sensitivity analysis of manufacturer protocol followed: bioMerieux (a), Roche TaqMan SPEX (b); and over time: Abbott 2-spot (c), bioMerieux (d), Roche TaqMan SPEX (e). (TIFF)

S1 Text HIV viral load meta-analysis study protocol. (PDF)

## Abbreviations

ART: antiretroviral treatment
CROI: Conference on Retroviruses and Opportunistic Infections
FVE: free virus elution
HSROC: hierarchical summary receiver-operator curve
IAS: International AIDS Society
ICASA: International Conference on AIDS and STIs in Africa
PRISMA: Preferred Reporting Items for Systematic Reviews and Meta-Analyses
QUADAS-2: Quality Assessment of Diagnostic Accuracy Studies
STARD: Standards for Reporting Studies of Diagnostic Accuracy

## References

[1] UNAIDS. Ending AIDS: Progress towards the 90-90-90 targets. 2017.

[2] Cohen MS, Chen YQ, McCauley M, Gamble T, Hosseinipour M, Kumarasamy N, et al. Prevention of HIV-1 infection with early antiretroviral therapy. N Engl J Med. 2011;365:493–505. doi: 10.1056/NEJMoa1105243 21767103 PMC3200068

[3] Gray RH, Wawer MJ, Brookmeyer R, Sewankambo NK, Serwadda D, Wabwire-Mangen F, et al. Probability of HIV-1 transmission per coital act in monogamous, heterosexual, HIV-1-discordant couples in Rakai, Uganda. Lancet. 2001;357:1149–1153. 11323041 10.1016/S0140-6736(00)04331-2

[4] Gray RH, Li X, Wawer MJ, Gange SJ, Serwadda D, Sewankambo NK, et al. Stochastic simulation of the impact of antiretroviral therapy and HIV vaccines on HIV transmission; Rakai, Uganda. IDS. 2003;17:1941–1951. doi: 10.1097/00002030-200309050-00013 12960827

[5] Rodger A, Cambiano V, Bruun T, Vernazza P, Collins S, Degen O, et al. Risk of HIV transmission through condomless sex in serodifferent gay couples with the HIV-positive partner taking suppressive antiretroviral therapy (PARTNER): final results of a multicentre, prospective, observational study. Lancet. 2019;393(10189):2428–2438. doi: 10.1016/S0140-6736(19)30418-0 31056293 PMC6584382

[6] Anema A, Lima VD, Johnston K, Levy A, Montaner JS. Expanded Highly Active Antiretroviral Therapy Coverage—A Powerful Strategy to Curb Progression to AIDS, Death and New Infections. Eur Infect Dis. 2009;3:41–43. 21243116 PMC3020076

[7] ART-LINC of IeDEA Group, Keiser O, Tweya H, Boulle A, Braitstein P, Schecter M, et al. Switching to second-line antiretroviral therapy in resource-limited settings: comparison of programmes with and without viral load monitoring. AIDS. 2009;23:1867–1874. doi: 10.1097/QAD.0b013e32832e05b2 19531928 PMC2956749

[8] HIV Surrogate Marker Collaborative Group. Human immunodeficiency virus type 1 RNA level and CD4 count as prognostic markers and surrogate end points: a meta-analysis. AIDS Res Hum Retroviruses. 2000;16:1123–1133. 10954887 10.1089/088922200414965

[9] Marschner IC, Collier AC, Coombs RW, D’Aquila RT, DeGruttola V, Fischl MA, et al. Use of changes in plasma levels of human immunodeficiency virus type 1 RNA to assess the clinical benefit of antiretroviral therapy. J Infect Dis. 1998;177:40–47. doi: 10.1086/513823 9419168

[10] Mellors JW, Rinaldo CR, Gupta P, White RM, Todd JA, Kingsley LA. Prognosis in HIV-1 infection predicted by the quantity of virus in plasma. Science. 1996;272:1167–1170. doi: 10.1126/science.272.5265.1167 8638160

[11] Murray JS, Elashoff MR, Iacono-Connors LC, Cvetkovich TA, Struble KA. The use of plasma HIV RNA as a study endpoint in efficacy trials of antiretroviral drugs. AIDS. 1999;13:797–804. doi: 10.1097/00002030-199905070-00008 10357378

[12] Sigaloff KC, Hamers RL, Wallis CL, Kityo C, Siwale M, Ive P, et al. Unnecessary antiretroviral treatment switches and accumulation of HIV resistance mutations; two arguments for viral load monitoring in Africa. J Acquir Immune Defic Syndr. 2011;58:23–31. doi: 10.1097/QAI.0b013e318227fc34 21694603

[13] Thiébaut R, Morlat P, Jacqmin-Gadda H, Neau D, Mercie P, Dabis F, et al. Clinical progression of HIV-1 infection according to the viral response during the first year of antiretroviral treatment. Groupe d’Epidémiologie du SIDA en Aquitaine (GECSA). AIDS. 2000;14:971–978. doi: 10.1097/00002030-200005260-00008 10853978

[14] Kantor R, Diero L, Delong A, Kamle L, Muyonga S, Mambo F, et al. Misclassification of first-line antiretroviral treatment failure based on immunological monitoring of HIV infection in resource-limited settings. Clin Infect Dis. 2009;49:454–462. doi: 10.1086/600396 19569972 PMC4859427

[15] Keiser O, MacPhail P, Boulle A, Wood R, Schechter M, Dabis F, et al. Accuracy of WHO CD4 cell count criteria for virological failure of antiretroviral therapy. Trop Med Int Health. 2009;14:1220–1225. doi: 10.1111/j.1365-3156.2009.02338.x 19624478 PMC3722497

[16] Moore DM, Awor A, Downing R, Kaplan J, Montaner JS, Hancock J, et al. CD4+ T-cell count monitoring does not accurately identify HIV-infected adults with virologic failure receiving antiretroviral therapy. J Acquir Immune Defic Syndr. 2008;49:477–484. doi: 10.1097/QAI.0b013e318186eb18 18989232

[17] Rawizza HE, Chaplin B, Meloni ST, Eisen G, Rao T, Sankale J-L, et al. Immunologic criteria are poor predictors of virologic outcome: implications for HIV treatment monitoring in resource-limited settings. Clin Infect Dis. 2011;53:1283–1290. doi: 10.1093/cid/cir729 22080121 PMC3246873

[18] World Health Organization. Consolidated guidelines on the use of antiretroviral drugs for treating and preventing HIV infection: recommendations for a public health approach. 2013. 24716260

[19] Abravaya K, Huang S, Erickson B, Mak WB. The use of dried blood spots with the Abbott RealTime HIV-1 viral load assay. XVII International AIDS Conference: Abstract no. CDB0034; Mexico City, Mexico: 2008.

[20] Aitken S, Kliphuis A, Bronze M, Wallis C, Kityo C, Balinda S, et al. Development and evaluation of an affordable real-time qualitative assay for determining HIV-1 virological failure in plasma and dried blood spots. J Clin Microbiol. 2013;51:1899–1905. doi: 10.1128/JCM.03305-12 23596235 PMC3716048

[21] Alvarez-Muñoz MT, Zaragoza-Rodríguez S, Rojas-Montes O, Palacios-Saucedo G, Vazquez-Rosales G, Gomez-Delgado A, et al. High correlation of human immunodeficiency virus type-1 viral load measured in dried-blood spot samples and in plasma under different storage conditions. Arch Med Res. 2005;36:382–386. doi: 10.1016/j.arcmed.2005.03.010 15950079

[22] Andreotti M, Pirillo M, Guidotti G, Ceffa S, Paturzo G, Germano P, et al. Correlation between HIV-1 viral load quantification in plasma, dried blood spots, and dried plasma spots using the Roche COBAS Taqman assay. J Clin Virol. 2010;47:4–7. doi: 10.1016/j.jcv.2009.11.006 19962936

[23] Arredondo M, Garrido C, Parkin N, Zahonero N, Bertagnolio S, Soriano V, et al. Comparison of HIV-1 RNA measurements obtained by using plasma and dried blood spots in the automated Abbott Real-Time viral load assay. J Clin Microbiol. 2012;50:569–572. doi: 10.1128/JCM.00418-11 22170904 PMC3295109

[24] Ayele W, Schuurman R, Messele T, Dorigo-Zetsma W, Mengistu Y, Goudsmit J, et al. Use of dried spots of whole blood, plasma, and mother’s milk collected on filter paper for measurement of Human Immunodeficiency Virus Type 1 burden. J Clin Microbiol. 2007;45:891–896. doi: 10.1128/JCM.01919-06 17251400 PMC1829137

[25] Brambilla D, Jennings C, Aldrovandi G, Bremer J, Comeau A, Cassol SA, et al. Multicenter evaluation of use of dried blood and plasma spot specimens in quantitative assays for Human Immunodeficiency Virus RNA: measurement, precision, and RNA stability. J Clin Microbiol. 2003;41:1888–1893. doi: 10.1128/JCM.41.5.1888-1893.2003 12734222 PMC154666

[26] Carmona S, Seiverth B, Horsfield P, eSa B, Goerdes M, Stevens W. Evaluation of COBAS AmpliPrep/COBAS TaqMan HIV-1 v2.0 test on dried blood spots. 2011; Rome, Italy: 2011.

[27] David S, Sachithanandham J, Jerobin J, Parasuram S, Kannangai R. Comparison of HIV-1 RNA level estimated with plasma and DBS samples: a pilot study from India (South). Indian J Med Microbiol. 2012;30:403–406. doi: 10.4103/0255-0857.103759 23183463

[28] Erba F, Brambilla D, Ceffa S, Ciccacci F, Luhanga R, Sidumo Z, et al. Measurement of viral load by the automated Abbott real-time HIV-1 assay using dried blood spots collected and processed in Malawi and Mozambique. S Afr Med J. 2015;105(12):10361038. doi: 10.7196/SAMJ.2015.v105i12.9673 26792161

[29] Fajardo E, Metcalf C, Chaillet P, Aleixo L, Pannus P, Panunzi I, et al. Prospective evaluation of diagnostic accuracy of dried blood spots from finger prick samples for determination of HIV-1 load with the NucliSENS Easy-Q HIV-1 Version 2.0 assay in Malawi. J Clin Microbiol. 2014;52:1343–1351. doi: 10.1128/JCM.03519-13 24501032 PMC3993687

[30] Fiscus SA, Brambilla D, Grosso L, Schock J, Cronin M. Quantitation of human immunodeficiency virus type 1 RNA in plasma by using blood dried on filter paper. J Clin Microbiol. 1998;36:258–260. doi: 10.1128/JCM.36.1.258-260.1998 9431960 PMC124847

[31] Garrido C, Zahonero N, Corral A, Arredondo M, Soriano V, De Mendoza C. Correlation between Human Immunodeficiency Virus Type 1 (HIV-1) RNA measurements obtained with dried blood spots and those obtained with plasma by use of Nuclisens EasyQ HIV-1 and Abbott RealTime HIV load tests. J Clin Microbiol. 2009;47:1031–1036. 19193847 10.1128/JCM.02099-08 PMC2668340

[32] Ikomey G, Atashili J, Okomo-Assoumou M, Mesembe M, Ndumbe P. Dried blood spots versus plasma for the quantification of HIV-1 RNA using the manual (PCR-ELISA) Amplicor Monitor HIV-1 Version 1.5 Assay in Yaounde, Cameroon. J Int Assoc Physicians AIDS Care (Chic). 2009;8:181–184. doi: 10.1177/1545109709333111 19357423

[33] Johannessen A, Garrido C, Zahonero N, Sandvik L, Naman E, Kivuyo SL, et al. Dried blood spots perform well in viral load monitoring of patients who receive antiretroviral treatment in rural Tanzania. Clin Infect Dis. 2009;49:976–981. doi: 10.1086/605502 19663598

[34] Kane CT, Ndiaye HD, Diallo S, Ndiaye I, Wade AS, Diaw PA, et al. Quantitation of HIV-1 RNA in dried blood spots by the real-time NucliSENS EasyQ HIV-1 assay in Senegal. J Virol Methods. 2008;148:291–295. doi: 10.1016/j.jviromet.2007.11.011 18242718

[35] Leelawiwat W, Young NL, Chaowanachan T, Ou CY, Culnane M, Vanprapa N, et al. Dried blood spots for the diagnosis and quantitation of HIV-1: stability studies and evaluation of sensitivity and specificity for the diagnosis of infant HIV-1 infection in Thailand. J Virol Methods. 2009;155:109–117. doi: 10.1016/j.jviromet.2008.09.022 18952125

[36] Lira R, Valdez-Salazar H, Vazquez-Rosales G, Rojas-Montes O, Ruiz-Tachiquin M, Torres-Ibarra R, et al. Genotypic testing for HIV-1 drug resistance using dried blood samples. Arch Virol. 2010;155:1117–1125. doi: 10.1007/s00705-010-0696-y 20496089

[37] Lofgren S, Morrissey A, Chevallier C, Malabeja A, Edmonds S, Amos B, et al. Evaluation of a dried blood spot HIV-1 RNA program for early infant diagnosis and viral load monitoring at rural and remote healthcare facilities. AIDS. 2009;23:2459–2466. doi: 10.1097/QAD.0b013e328331f702 19741481 PMC2890230

[38] Marconi A, Balestrieri M, Comastri G, Pulvirenti FR, Gennari W, Tagliazucchi S, et al. Evaluation of the Abbott Real-Time HIV-1 quantitative assay with dried blood spot specimens. Clin Microbiol Infect. 2009;15:93–97. doi: 10.1111/j.1469-0691.2008.02116.x 19220340

[39] Mbida AD, Sosso S, Flori P, Saoudin H, Lawrence P, Monny-Lobe M, et al. Measure of viral load by using the Abbott Real-Time HIV-1 assay on dried blood and plasma spot specimens collected in 2 rural dispensaries in Cameroon. J Acquir Immune Defic Syndr. 2009;52:9–16. doi: 10.1097/QAI.0b013e3181aeccbc 19620878

[40] Mercier-Delarue S, Vray M, Plantier J, Maillard T, Adjout Z, de Olivera F, et al. Higher specificity of nucleic acid sequence-based amplification isothermal technology than of real-time PCR for quantification of HIV-1 RNA on dried blood spots. J Clin Microbiol. 2014;52:52–56. doi: 10.1128/JCM.01848-13 24131691 PMC3911453

[41] Monleau M, Butel C, Delaporte E, Boillot F, Peeters M. Effect of storage conditions of dried plasma and blood spots on HIV-1 RNA quantification and PCR amplification for drug resistance genotyping. J Antimicrob Chemother. 2010;65:1562–1566. doi: 10.1093/jac/dkq205 20542904

[42] Monleau M, Aghokeng A, Eymard-Duvernay S, Dagnra A, Kania D, Ngo-Giang-Huong N, et al. Field evaluation of dried blood spots for routine HIV-1 viral load and drug resistance monitoring in patients receiving antiretroviral therapy in Africa and Asia. J Clin Microbiol. 2014;52:578–586. doi: 10.1128/JCM.02860-13 24478491 PMC3911301

[43] Mtapuri-Zinyowera S, Taziwa F, Metcalf C, Mbofana E, De Weerdt S, Flevaud L, et al. Field evaluation of performance of dried blood spots (DBS) from finger-prick for the determination of viral load in a resource-constrained setting in urban and rural Zimbabwe. 7th IAS Conference on HIV Pathogenesis and Treatment and Prevention: Abstract no. TUPE271. Kuala Lumpur, Malaysia; 2013.

[44] Mwaba P, Cassol S, Nunn A, Pilon R, Chintu C, Janes M, et al. Whole blood versus plasma spots for measurement of HIV-1 viral load in HIV-infected African patients. Lancet. 2003;362:2067–2068. doi: 10.1016/S0140-6736(03)15103-3 14697808

[45] Neogi U, Gupta S, Rodridges R, Sahoo PN, Rao SD, Rewari BB, et al. Dried blood spot HIV-1 RNA quantification: a useful tool for viral load monitoring among HIV-infected individuals in India. Indian J Med Res. 2012;136:956–962. 23391790 PMC3612324

[46] Ngwende S, Mudenge B, Madzimure D, Mudenge G. Evaluation of the use of dried blood spots for viral load monitoring in resource-limited settings in Zimbabwe. 2012; Washington, DC, USA: 2012.

[47] Onkendi E, Abuya D, Mumo R, King-wara L, Kangogo GK, Bera SK, et al. Evaluation of dried blood spots as an alternative sample type in HIV-1 RNA quantification from patients receiving anti-retroviral treatment in Kenya. Clin Med Diagn. 2017;7:75–79.

[48] Ouma K, Basavaraju S, Okonji J, Williamson J, Thomas T, Mills L, et al. Evaluation of quantification of HIV-1 RNA viral load in plasma and dried blood spots by use of the semiautomated Cobas Amplicor assay and the fully automated Cobas Ampliprep/TaqMan assay, Version 2.0, in Kisumu, Kenya. J Clin Microbiol. 2013;51:1208–1218. doi: 10.1128/JCM.03048-12 23390278 PMC3666812

[49] Pannus P, Claus M, Perez Gonzalez MM, Ford N, Fransen K. Sensitivity and specificity of dried blood spots for HIV-1 viral load quantification. Medicine. 2016;95:e5475. 27902602 10.1097/MD.0000000000005475 PMC5134769

[50] Pirillo M, Recordon-Pinson P, Andreotti M, Mancini M, Amici R, Giuliano M. Quantification of HIV-RNA from dried blood spots using the Siemens VERSANT(R) HIV-1 RNA (kPCR) assay. J Antimicrob Chemother. 2011;66:2823–2826. 21930572 10.1093/jac/dkr383

[51] Pollack T, Duong H, Truong P, Pham TT, Do CD, Colby D. Sensitivity and specificity of two dried blood spot methods for HIV-1 viral load monitoring among patients in Hanoi, Vietnam. PLoS ONE. 2018;13:e0191411. doi: 10.1371/journal.pone.0191411 29346431 PMC5773210

[52] Reigadas S, Schrive MH, Aurillac-Lavignolle V, Fleury HJ. Quantitation of HIV-1 RNA in dried blood and plasma spots. J Virol Methods. 2009;161:177–180. doi: 10.1016/j.jviromet.2009.06.002 19523984

[53] Rottinghaus E, Ugbena R, Diallo K, Bassey O, Azeez A, Dezos J, et al. Dried blood spot specimens are a suitable alternative sample type for HIV-1 viral load measurement and drug resistance genotyping in patients receiving first-line antiretroviral therapy. Clin Infect Dis. 2012;54:1187–1195. doi: 10.1093/cid/cis015 22412066 PMC11528918

[54] Rutstein SE, Kamwendo D, Lugali L, Thengolose I, Tegha G, Fiscus SA, et al. Measures of viral load using Abbott RealTime HIV-1 Assay on venous and fingerstick dried blood spots from provider-collected specimens in Malawian District Hospitals. J Clin Virol. 2014;60(4):392–398. doi: 10.1016/j.jcv.2014.05.005 24906641 PMC4073118

[55] Sahoo MK, Varghese V, White E, Winslow M, Katzenstein DA, Shafer RW, et al. Evaluation of the Aptima HIV-1 Quant Dx assay using plasma and dried blood spots. J Clin Microbiol. 2016;54:2597–2601. doi: 10.1128/JCM.01569-16 27535684 PMC5035416

[56] Sawadogo S, Shiningavamwe A, Chang J, Maher A, Zhang G, Yang C, et al. Limited utility of dried-blood- and plasma spot-based screening for antiretroviral treatment failure with Cobas Ampliprep/TaqMan HIV-1 Version 2.0. J Clin Microbiol. 2014;52:3878–3883. doi: 10.1128/JCM.02063-14 25143579 PMC4313218

[57] Schmitz M, Agolory S, Junghae M, Broyles LN, Kimeu M, Ombayo J, et al. Field evaluation of dried blood spots for HIV-1 viral load monitoring in adults and children receiving antiretroviral treatment in Kenya: Implications for scale-up in resource-limited settings. J Acquir Immune Defic Syndr. 2017;74:399–406. doi: 10.1097/QAI.0000000000001275 28002185

[58] Taieb F, Hong T, Ho H. First field evaluation of the optimized CE marked Abbott protocol for HIV RNA testing on dried blood spot in a routine clinical setting in Vietname. PLoS ONE. 2018;13:e01901920. 10.1371/journal.pone.0191920 PMC5806875 29425216

[59] Tang N, Pahalawatta V, Frank A, Bagley Z, Viana R, Lampinen J, et al. HIV-1 viral load measurement in venous blood and fingerprick blood using Abbott RealTime HIV-1 DBS assay. J Clin Virol. 2017;92:56–61. doi: 10.1016/j.jcv.2017.05.002 28531553

[60] Tariro Makadzange A, Boyd F, Chimukangara B, Masimirembwa C, Katzenstein D, Ndhlovu CE. A simple phosphate-buffered-saline-based extraction method improves specificity of HIV viral load monitoring using dried blood spots. J Clin Microbiol. 2017;55:2172–2179. doi: 10.1128/JCM.00176-17 28468852 PMC5483919

[61] Uttayamakul S, Likanonsakul S, Sunthornkachit R, Kuntiranont K, Louisirirotchanakul S, Chaovavanich A, et al. Usage of dried blood spots for molecular diagnosis and monitoring HIV-1 infection. J Virol Methods. 2005;128:128–134. doi: 10.1016/j.jviromet.2005.04.010 15913797

[62] van Deursen P, Oosterlaken T, Andre P, Verhoeven A, Bertens L, Trabaud MA, et al. Measuring human immunodeficiency virus type 1 RNA loads in dried blood spot specimens using NucliSENS EasyQ HIV-1 v2.0. J Clin Virol. 2010;47:120–125. doi: 10.1016/j.jcv.2009.11.021 20018560

[63] Vidya M, Saravanan S, Rifkin S, Solomon SS, Waldrop G, Mayer KH, et al. Dried blood spots versus plasma for the quantitation of HIV-1 RNA using a real-Time PCR, m2000rt assay. J Virol Methods. 2012;181:177–181. doi: 10.1016/j.jviromet.2012.02.006 22401801

[64] Viljoen J, Gampini S, Danaviah S, Valea D, Pillay S, Kania D, et al. Dried blood spot HIV-1 RNA quantification using open real-time systems in South Africa and Burkina Faso. J Acquir Immune Defic Syndr. 2010;55:290–298. doi: 10.1097/QAI.0b013e3181edaaf5 20700058

[65] Waters L, Kambugu A, Tibenderana H, Meya D, John L, Mandalia S, et al. Evaluation of filter paper transfer of whole-blood and plasma samples for quantifying HIV RNA in subjects on antiretroviral therapy in Uganda. J Acquir Immune Defic Syndr. 2007;46:590–593. 18193501 10.1097/qai.0b013e318159d7f4

[66] Wu X, Crask M, Ramirez H, Landas T, Do TD, Honisch C, et al. A simple method to elute cell-free HIV from dried blood spots improves their usefulness for monitoring therapy. J Clin Virol. 2015;65:38–40. doi: 10.1016/j.jcv.2015.01.022 25766985

[67] Yapo V, Toni TD, Desmonde S, Amani-Bosse C, Oga M, Lenaud S, et al. Evaluation of dried blood spot diagnosis using HIV1-DNA and HIV1-RNA Biocentric assays in infants in Abidjan, Côte d’Ivoire. The Pedi-Test DBS ANRS 12183 Study. J Virol Methods. 2013;193:439–445. doi: 10.1016/j.jviromet.2013.07.011 23872283

[68] Yek C, Massanella M, Peling T, Lednovich K, Nair SV, Worlock A, et al. Evaluation of the Aptima HIV-1 Quant Dx assay for HIV-1 RNA quantitation in different biological specimen types. J Clin Microbiol. 2017;55:2544–2553. doi: 10.1128/JCM.00425-17 28592548 PMC5527433

[69] Zeh C, Ndlege K, Inzaule S, Achieng R, Williamson J, Chang JC-W, et al. Evaluation of the performance of Abbott m2000 and Roche COBAS Ampliprep/COBAS Taqman assays for HIV-1 viral load determination using dried blood spots and dried plasma spots in Kenya. PLoS ONE. 2017;12:e0179316. doi: 10.1371/journal.pone.0179316 28622370 PMC5473550

[70] Moher D, Liberati A, Tetzlaff J, Altman D. Preferred Reporting Items for Systematic Reviews and Meta-Analyses: The PRISMA Statement. PLoS Med. 2009;6:e1000097. doi: 10.1371/journal.pmed.1000097 19621072 PMC2707599

[71] Bossuyt PM, Reitsma JB, Bruns DE, Gatsonis CA, Glasziou PP, Irwig LM, et al. Towards complete and accurate reporting of studies of diagnostic accuracy: the STARD initiative. BMJ. 2003;326:41–44. doi: 10.1136/bmj.326.7379.41 12511463 PMC1124931

[72] Whiting PF, Rutjes AW, Westwood ME, Mallett S, Deeks JJ, Reitsma JB, et al. QUADAS-2: a revised tool for the quality assessment of diagnostic accuracy studies. Ann Intern Med. 2011;155:529–536. doi: 10.7326/0003-4819-155-8-201110180-00009 22007046

[73] Takkouche B, Cadarso-Suárez C, Spiegelman D. Evaluation of old and new tests of heterogeneity in epidemiologic meta-analysis. Am J Epidemiol. 1999;150:206–215. doi: 10.1093/oxfordjournals.aje.a009981 10412966

[74] Dahabreh IJ, Trikalinos TA, Lau J, Schmid C. An empirical assessment of bivariate methods for meta-analysis of test accuracy. Methods Research Reports. 2012;12. 23326899

[75] Pasternak A, Jurriaans S, Bakker M, Prins J, Berkhout B, Lukashov V. Cellular levels of HIV unspliced RNA from patients on combination antiretroviral therapy with undetectable plasma viremia predict the therapy outcome. PLoS ONE. 2009;4:e8490. doi: 10.1371/journal.pone.0008490 20046870 PMC2795168

[76] Cobb BR, Vaks JE, Do T, Vilchez RA. Evolution in the sensitivity of quantitative HIV-1 viral load tests. J Clin Virol. 2011;52(Suppl 1):S77–S82. doi: 10.1016/j.jcv.2011.09.015 22036041

[77] Jennings C, Harty B, Granger S, Wager C, Crump J, Fiscus SA, et al. Cross-platform analysis of HIV-1 RNA data generated by a multicenter assay validation study with wide geographic representation. J Clin Microbiol. 2012;50:2737–2747. doi: 10.1128/JCM.00578-12 22692747 PMC3421531

[78] World Health Organization. Technical and operational considerations for implementing HIV viral load testing: interim technical update. 2014.

[79] World Health Organization. Consolidated guidelines on the use of antiretroviral drugs for treating and preventing HIV infection: recommendations for a public health approach. 2016. 27466667

[80] Piatak M, Saag MS, Yang LC, Clark SJ, Kappes JC, Luk K-C, et al. High levels of HIV-1 in plasma during all stages of infection determined by competitive PCR. Science. 1993;259:1749–1754. doi: 10.1126/science.8096089 8096089

[81] Hamers RL, Smit P, Stevens W, Schuurman R, Rinke de Wit TF. Dried fluid spots for HIV type-1 viral load and resistance genotyping: a systematic review. Antivir Ther (Lond). 2009;14:619–629. 19704164

[82] Parkin NT. Measurement of HIV-1 viral load for drug resistance surveillance using dried blood spots: literature review and modeling of contribution of DNA and RNA. AIDS Rev. 2014;16:160–171. 25221990

[83] Smit P, Sollis K, Fiscus S, Ford N, Vitoria M, Essajee S, et al. Systematic review of the use of dried blood spots for monitoring HIV viral load and for early infant diagnosis. PLoS ONE. 2014;9:e86461. doi: 10.1371/journal.pone.0086461 24603442 PMC3945725

