# Supplementary figures and images for: The performance of using dried blood spot specimens for HIV-1 viral load testing: A systematic review and meta-analysis
Source: PLoS Med. 2022 Aug 22;19(8):e1004076. doi: 10.1371/journal.pmed.1004076 (PMC9447868; doi:10.1371/journal.pmed.1004076)

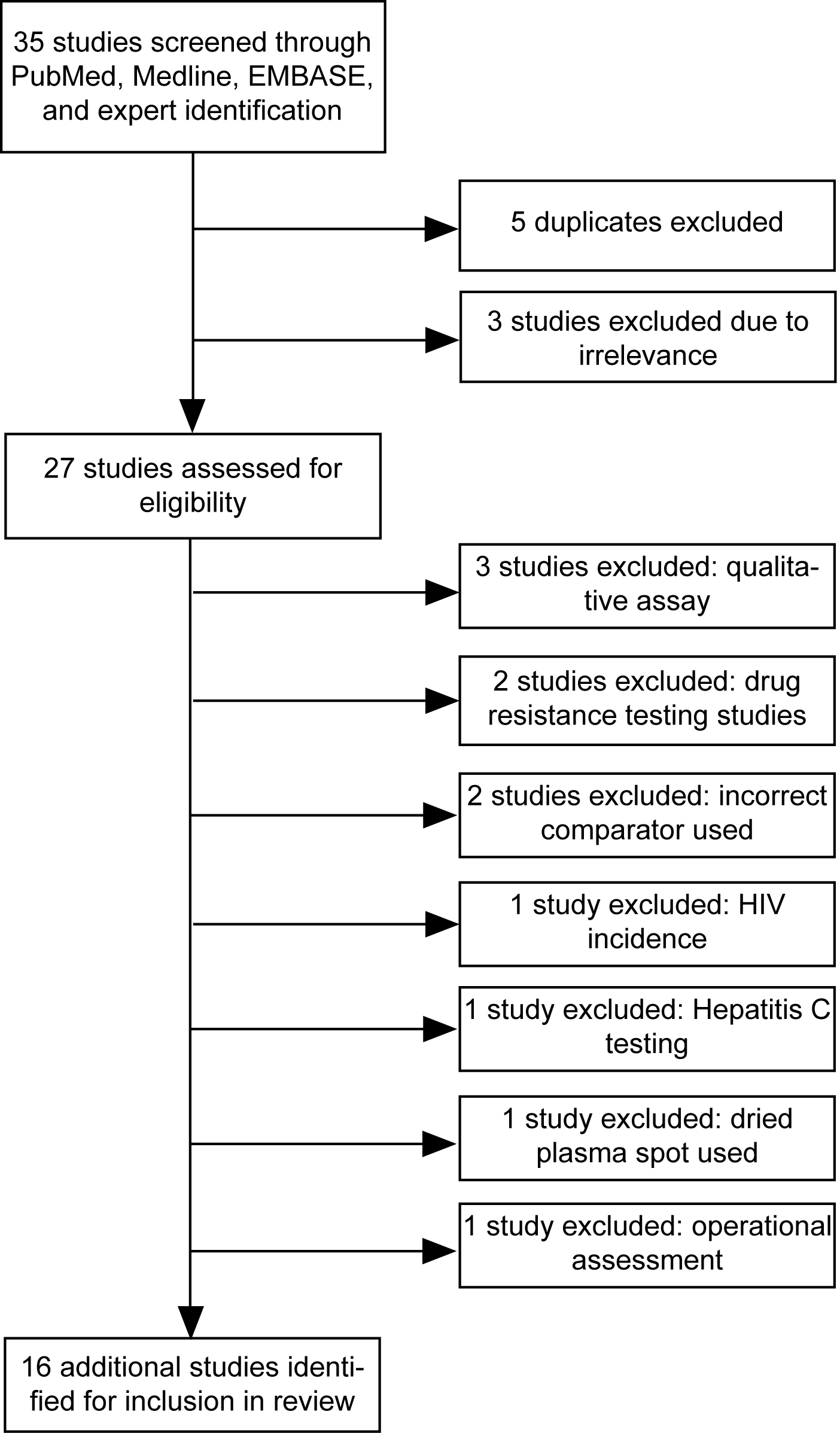

Supplement: S1 Fig — (TIF) [file pmed.1004076.s002.tif]

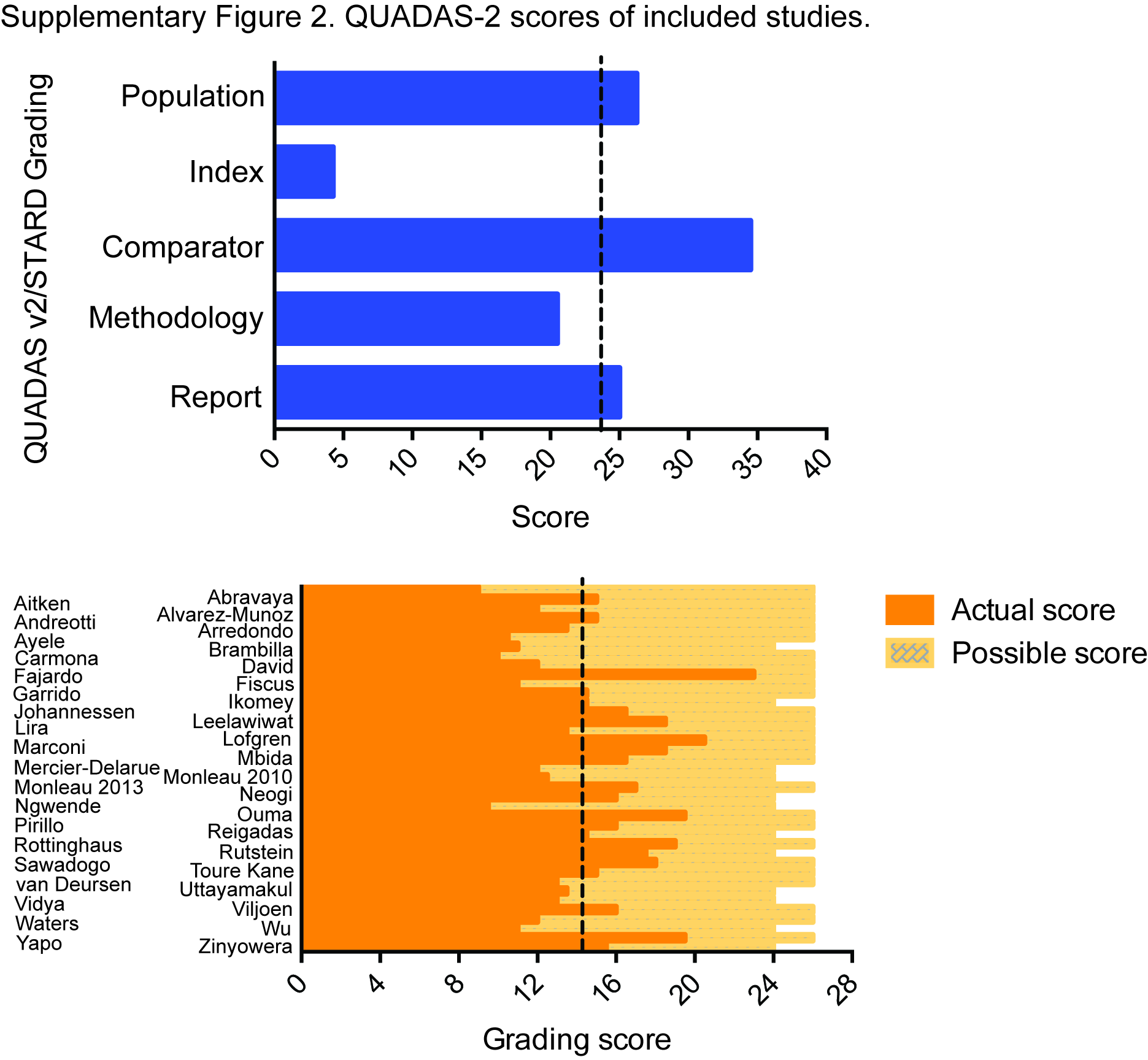

Supplement: S2 Fig — (TIF) [file pmed.1004076.s003.tif]

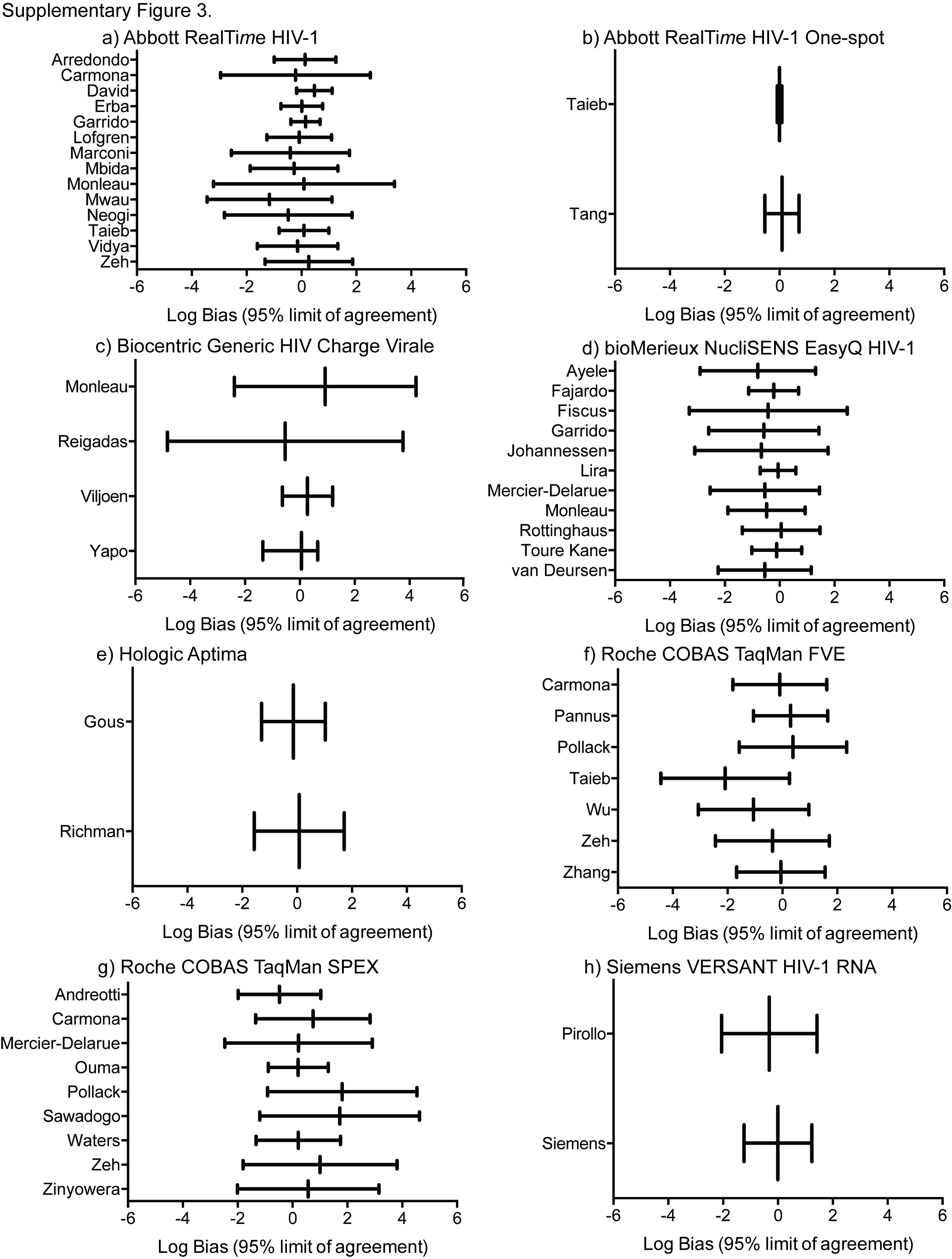

Supplement: S3 Fig — (TIF) [file pmed.1004076.s004.tif]

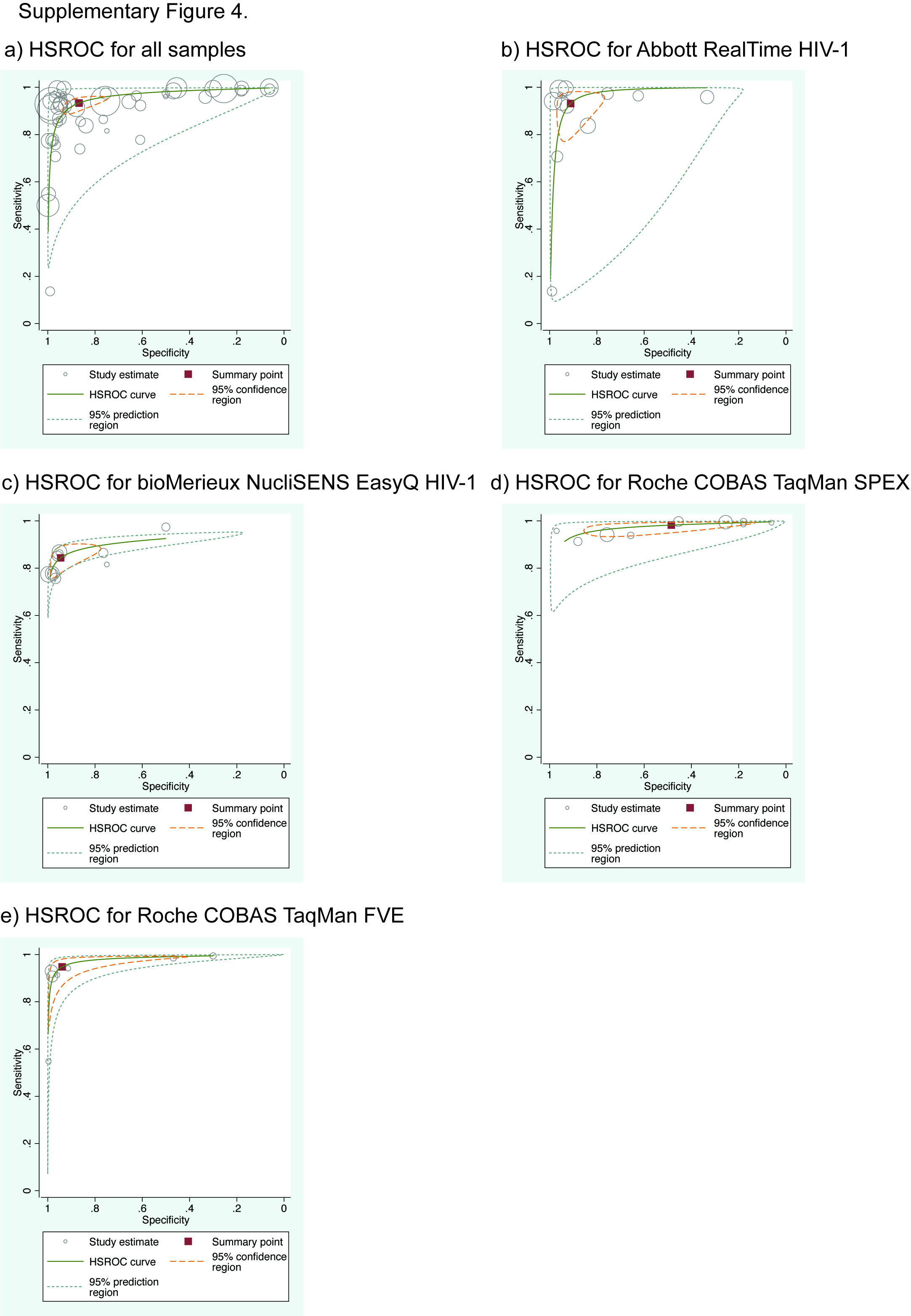

Supplement: S4 Fig — Circle sizes were relative to the study sample size. The red square was the pooled summary point. (TIF) [file pmed.1004076.s005.tif]

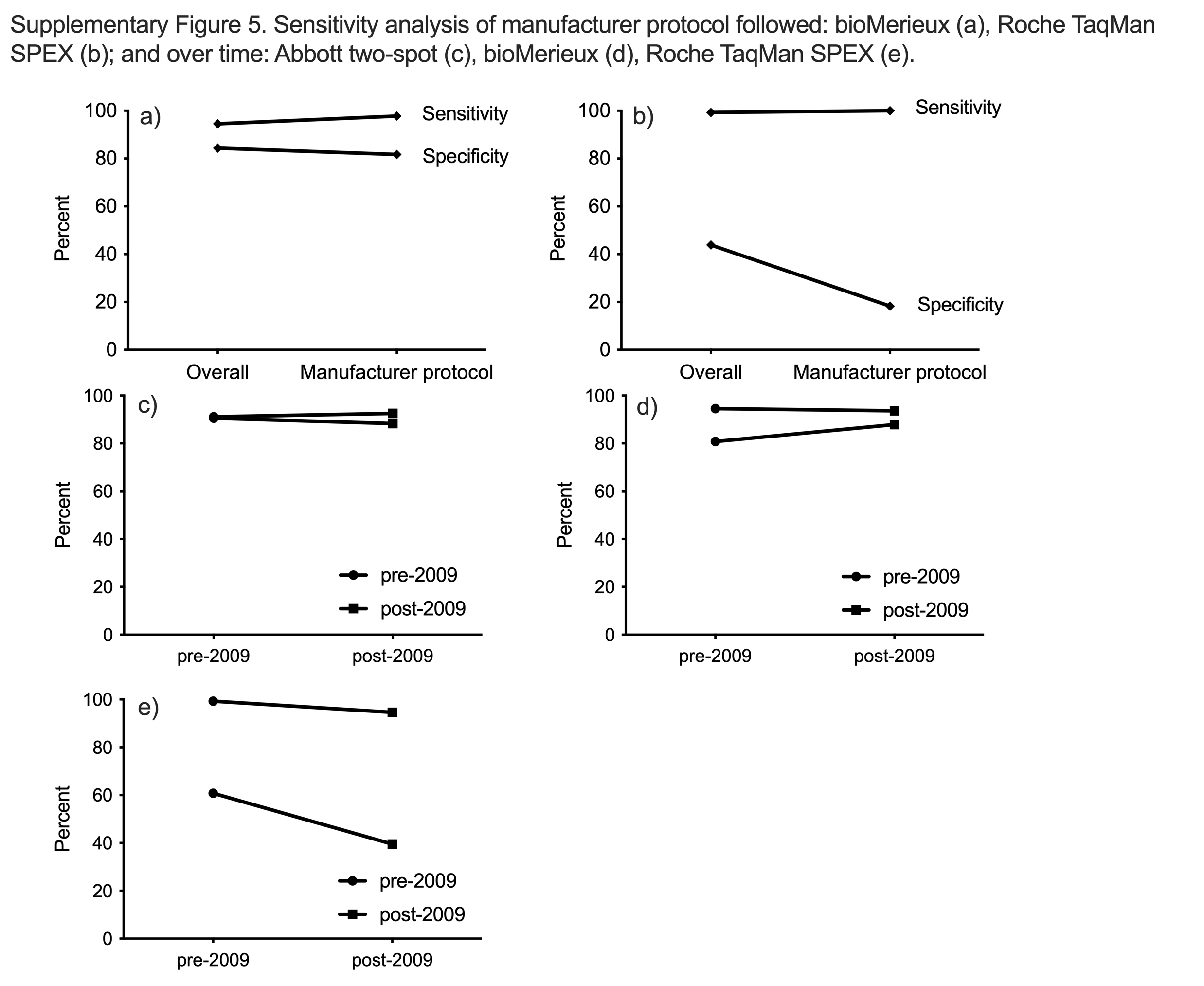

Supplement: S5 Fig — (TIFF) [file pmed.1004076.s006.tiff]
