# Supplementary material for: The performance of using dried blood spot specimens for HIV-1 viral load testing: A systematic review and meta-analysis
Source: PLoS Med. 2022 Aug 22;19(8):e1004076. doi: 10.1371/journal.pmed.1004076 (PMC9447868; doi:10.1371/journal.pmed.1004076)
Supplement: S1 Text — (PDF) [file pmed.1004076.s007.pdf]

Project protocol:

DBS and DPS Sample Collection for HIV Viral Load Testing: Meta – Analysis of the Available Technologies

October 2013

Study investigators

Chunfu Yang (US CDC)

Debrah Boeras (US CDC)

Wendy Stevens (RSA NHLS)

Sergio Carmona (RSA NHLS)

Lara Vojnov (CHAI)

Trevor Peter (CHAI)

1. Introduction

Viral load has been used in developed countries for many years as the standard of care to monitor patients' viral load, ART failure, and drug resistance emergence. Unfortunately the high costs, the necessity of highly specialized and well-equipped laboratories, and difficult sample collection logistics has slowed the uptake of viral load testing in developing countries. The widely-accepted 'gold standard' sample for viral load testing is plasma separated from EDTA anti-coagulated blood. The HIV viral load of plasma from HIV-positive patients has been correlated with several immunological and virological events as well as being an indicator of disease progression and/or status. Unfortunately, however, the storage and transportation time of whole blood at room temperature for viral load testing is limited to 24 hours or less, depending on the technology and most health care facilities lack centrifuges or refrigerators and freezers to separate plasma from blood themselves before shipment to the laboratory for testing.

2. Justification

Dried blood spot (DBS) samples for HIV testing were introduced a few years ago and are used primarily for early infant diagnosis by PCR. They are beneficial in resource-limited settings as they do not require centrifuges, refrigerators, or freezers at the sample collection site, can be stored and transported for weeks at room temperature, and require a simple finger-prick or heel-stick blood sample. Once delivered to the laboratory, simple sample processing is done before viral load analysis. The level of access to viral load can increase exponentially with the introduction of DBS sample collection. To date, there have been several studies conducted that compared viral load results from dried blood spot (DBS) samples and plasma separated from EDTA-anti-coagulated blood. While there is some variability in the results seen, it is clear that DBS has a higher limit of detection (approximately 3 log copies/ml) than plasma (generally less than 100 copies/ml). This is most likely due to the smaller input volume for DBS (25-50 ul) compared to approximately 1 ml of input volume for plasma.

Because a DBS sample is a drop or two of whole blood, some technologies detect pro-viral DNA and cell-based RNA in addition to RNA circulating in the plasma. While this additional detection may be negligible at higher, less clinically relevant viral loads, with WHO recommended ART failure threshold rates of 5,000 or 1,000 copies/ml, this can cause some over-quantification and subsequent misclassification of patients. This has been seen in some studies, such as those performing viral load testing on RT-PCR nucleic-acid based technologies. Interestingly, however, this over-quantification below 3 log copies/ml may not occur with NASBA technologies.

Dried plasma spot (DPS) samples are also being considered as sample collection types for viral load testing, but the performance is currently unclear.

Viral load testing is primarily performed on five platforms in sub-Saharan Africa: Abbott RealTime HIV-1, Biocentric, bioMerieux NucliSENS EasyQ HIV-1, Roche COBAS Ampliprep or TaqMan HIV-1 Test, and Siemens VERSANT kPCR Molecular System. The technical performance standard of each technology with DBS as the sample collection method is, unfortunately, unclear. **We are, therefore, proposing to conduct a meta-analysis of viral load data comparing results from DBS and DPS samples with those from plasma on each of the five viral load technologies to better understand the performance and limitations of each**

technology when used with DBS and DPS samples and attempt to find a solution to the variability and over-quantification observed. Doing so should provide guidance to country programs looking to implement and expand access to viral load testing.

3. Expected results

We expect this research will provide a comprehensive review of the performance of using DBS sample specimens for laboratory-based viral load testing. The analysis will show overall performance accuracy and precision of DBS and DPS samples to plasma extracted from whole blood based on various accuracy tests explained the methodology section. The results will give guidance on the use of DBS and DPS samples for viral load testing in different clinical settings and with each available viral load technology.

Study objectives

This study aims to:

Identify, collate and summarize multiple DBS and DPS viral load evaluations in order to:

- Assess the statistical characteristics and trends of each viral load reference technology through sensitivity, specificity, PPV, NPV, agreement, etc.
- Use aggregated data to assess the clinical utility of DBS and DPS for viral load testing, including misclassification rates.
- Determine whether a mathematical correction is necessary and possible when using DBS and DPS as the sample method for ART failure monitoring specific for each viral load reference technology.
- Provide recommendations for the appropriate use of DBS and DPS samples for viral load testing using the five most prevalent technologies.

4. Methodology

4.1 Study design

It is estimated that at least 35 independent studies across different countries and clinical settings have assessed the accuracy of using DBS and DPS samples for viral load testing to date. We aim to collate data from these studies and analyze cross-sectional observational primary data by cohort and technology. Each study will use observation level data with at least one measurement of viral load result by the reference lab technology using plasma and one viral load result using DBS and DPS samples. Data will be compared by each of the five primary viral load technologies as well as, if possible, by patients on ART.

Study search

Study terms to be used include: “dried blood spot”, “dried plasma spot”, “plasma”, “technical performance”, “comparison”, “evaluation”, “viral load testing” and each of the technology names as included above. The study search will include the following databases: PubMed, Google Scholar, Cochrane Central Register of Controlled Trials, EMBASE, LILACS, Ovid MEDLINE, WHOLIS, ADOLEC, MedCarib, IBACS, Web of knowledge and WHO Global Index Medicus, the International Clinical Trials Registry Platform, etc.

Conference abstracts will be searched using the Conference on Retroviruses and Opportunistic Infections (CROI) and International AIDS Society (IAS) conference websites. Bibliographies of screened and selected studies as well as review articles will be reviewed for possible inclusion. Finally, we will contact content experts to obtain further studies, including unpublished studies. When possible titles will be screened and selected in duplicate.

Studies will be included if they meet the following criteria:

- Technical evaluation data comparing dried blood spot or dried plasma spot specimens to plasma
- Technical evaluation pertaining to viral load testing
- Performed testing on HIV-positive blood

Studies will be excluded if they contain one of the following criteria:

- Perform viral load testing using spiked blood samples or panels
- Compared dried blood spot or dried plasma spot specimens to plasma with a different assay, such as drug resistance
- Performed a qualitative analysis of dried blood spot samples (ie. early infant diagnosis)
- The comparator or gold standard was a sample type other than plasma

Studies will be graded using the GRADE and STARD criteria for quality.

4.2 Summary of studies

Each of the studies will be summarized outlining the principle components of each cohort. The summary will include viral load technology used, sample size, test setting, patient characteristics, etc. A description of the study design and methods will be included. The findings will be summarized for the studies that have been included, published or unpublished.

4.3 Data Analysis

Overview – We are aiming to include data from several sources, both published and unpublished data. All partners, such as the CDC, MSF, national reference laboratories, and academic institutions will all be invited to share their data for inclusion in this meta-analysis. We will aim to collect primary viral load results obtained from HIV-positive patients with both a DBS (or DPS) and plasma sample on one of the five technologies noted above. We will compare the results and statistical analyses of each of the five technologies. The analysis will be based on scientific methods for comparisons of new laboratory technologies with a reference, or gold standard, approach. The data will be imported into Microsoft Excel and analyzed by GraphPad Prism, Stata 13, and SAS. Separate analyses will be performed for DBS and DPS sample types.

The data collected will be analyzed in several ways:

- Bland-Altman analysis with bias (and standard deviation) and 95% limits of agreement for the entire dataset and broken down by each technology;

- Regression analysis with R^2 analysis for the entire dataset and broken down by each technology;
- Misclassification rates for the entire dataset and broken down by each technology; additionally, we will analyze the misclassification rates at different ART failure threshold values for the DBS sample;
- Sensitivity, specificity, positive predictive value, negative predictive value, overall agreement, and Kappa value at different ART failure thresholds for the entire dataset and broken down by each technology;
- Concordance, discordance, bias (and standard deviation) and 95% limits of agreement for the entire dataset and broken down by each technology; additionally, we will analyze these metrics at different ART failure threshold values for the DBS or DPS sample;
- Mathematical analysis to better understand the relationship between DBS or DPS and plasma below 4 log copies/ml.

Bias

This will determine the amount of difference the DBS or DPS sample collection differs from the gold standard using various measures. This will take the primary data and may be conducted using:

Bland-Altman plot - will be used to assess bias and 95% limits of agreement of DBS or DPS sample results compared with plasma sample results. This analysis will look across studies, by technology, by cohort as well as for ART patients, if possible.

Plots - a regression scatter-plot will be used to look at the pairs of results. The plots will be on the actual values of each test, this will help to visualize the degree of dispersion and skewness and identify outliers as well as perform R^2 analysis. This analysis will look across studies, by technology, by cohort as well as for ART patients, if possible.

Misclassification

A second step will look at the proportion of patients that would have been falsely classified as either eligible for switching to 2nd line treatment or ineligible. The level of accuracy that is acceptable will be determined prior to analysis. The misclassification analysis will assess several ART switching thresholds, including the WHO 2010 recommendation of 5,000 copies/ml and WHO 2013 recommendation of 1,000 copies/ml. The misclassification will be presented as a percentage as the number of observations that would be classified incorrectly based on the ART switching thresholds. This will be measured if the DBS sample test result predicts "true" or "false". Using the example of a cutoff off of 5,000 copies/ml viral load as an ART switching threshold.

	DBS/DPS Result above 5,000 copies/ml	DBS/DPS Result below 5,000 copies/ml
Reference Result above 5,000 copies/ml	True positive (TP)	False negative (FN)
5,000 copies/ml	False positive (FP)	True negative (TN)

Test efficiency will be measured as the TP+TN/ TP+TN+FP+FN.

Upward misclassification – This is the percent of all observations that are greater than the threshold using DBS or DPS samples, but are below the threshold using plasma samples. For example, this would be the number of patients that received viral load results above 5,000 copies/ml using DBS or DPS samples and would have been eligible to switch to 2nd line treatment, however using plasma these specimens were found to have viral loads less than 5,000 copies/ml and would have remained on 1st line treatment. This is measured as the FP/ TP+TN+FP+FN.

Downward misclassification – alternatively, the downward misclassification estimates will show the percent of specimens that would have been misclassified as being below the threshold using DBS or DPS samples, but above the threshold using plasma. This is measured as the FN/ TP+TN+FP+FN.

Accuracy and Precision

Additional analyses to determine both accuracy and precision – further understanding of the accuracy and precision of DBS or DPS samples compared to plasma for viral load testing. This will take the primary data and may be conducted using:

Sensitivity, specificity, positive predictive value, negative predictive value, overall agreement and Kappa values. This analysis will look across studies, by technology, by cohort as well as for ART patients, if possible.

Concordance and discordance. This analysis will look across studies, by technology, by cohort as well as for ART patients, if possible.

All of the above statistical methods will be used on each cohort, for each technology as well as for each technology using data from patients on ART, if possible. Additionally study cohorts and data may be further analyzed by sample volume, DBS or DPS card used, time to testing and other study design specifics. Finally, other potential analyses may be included to identify if a mathematical correction is necessary and possible for technologies with poor performance.

Exploring and Accounting for heterogeneity between studies

Study heterogeneity may occur for multiple reasons including differences in sample size, study quality, study designs, and/or data collection methods. For this work, a series of methods will be used to first quantify and then account for the presence of any study heterogeneity. To determine the presence of between-study heterogeneity, the Q-statistic will first be calculated (Takakouche 1999). If there is a significant level of heterogeneity between the selected studies, all study-specific accuracy measures will then be plotted and visually evaluated for outliers. Additionally, a Galbraith plot will be used to see if there are particular studies which may require further examination before being used in the pooled summary measures (Lijmer 2002).

After confirming and exploring the presence of heterogeneity, bivariate and univariate random effects models will be used to estimate the pooled summary measures for bias, accuracy, and precision accounting for between-study variation. While pooling all the data assumes that all the studies were performed in the identical manner, the random effects approach relaxes this assumption, and therefore, provides summary estimates of diagnostic accuracy allowing for this variation. The models will be completed using METANDI commands in STATA 13.

Sensitivity Analyses

Sensitivity analyses will also be performed focusing on four primary sub-analyses:

1. Analyses including only the latest version and/or assay of each technology assay;
2. Analysis including only those studies that compiled exactly to manufacturer's instructions for processing the dried blood spot sample and extracting nucleic acids;
3. Analyses by specimen collection, specimen storage method, geography, and dried blood spot card type;
4. Analysis including only those patients known to be on antiretroviral therapy.

5. Ethical considerations

This study will not involve the collection of new information or samples from patients and does not have a clinical trial component. All data that will be used in this study will be gathered and used with permission from the study primary investigators. Only data that was collected with local standard approval processes will be used and no patient names or identification numbers will be used or shared. Data will be encrypted if requested by study primary investigators and will not be shared outside of the project team. Following completion of the study, data can be deleted from study files if requested. Data sharing agreements have been developed and will be signed if requested.

6. Timeline

Activities	Oct '13	Nov '13	Dec '13	Jan '14	Feb '14	Mar '14	Apr '14	May '14	Jun '14	Jul '14
Identify studies for inclusion	X	X								
Contact study PIs / data collection	X	X	X	X	X					
Conduct data analysis					X	X	X	X	X	X
Write up of report								X	X	X
Finalize report/ disseminate										X

7. Dissemination of Study Findings

The primary output of the research will be to provide guidance on the performance of DBS and DPS sample collection specimens for viral load testing. The study report will provide the findings as well as outline specific guidelines and recommendations. The report will be disseminated through standard channels in order to reach stakeholders as well as be published in an international peer-reviewed journal and scientific conferences. The study findings will also inform guideline documents on the use of DBS samples for viral

load testing. This will be published and disseminated within the health care community via standard mechanisms, including meetings, trainings, internet portals, Ministries of Health and development partners. Additionally, depending on the significance of the findings, a policy brief will be published on the ASLM website and disseminated accordingly.

Annex:
Template of Data Request Letter for the Dried Blood Spot Samples for Viral Load Testing Meta-Analysis. A similar letter will be sent to principal investigators using dried plasma spots.

Dried Blood Spot Samples for Viral Load Testing Meta-Analysis - Template Data Request Letter

Dear ***Principle Investigator X,***

We are contacting you in regards to the evaluation your institution conducted comparing Dried Blood Spot (DBS) samples with plasma for viral load testing. There have been several similar studies completed on a variety of viral load technologies (Roche, Abbott, bioMerieux, etc.). The United States Centers for Disease Control and Prevention (CDC), South African National Health Laboratory Service, Clinton Health Access Initiative (CHAI) and London School of Hygiene and Tropical Medicine are collaborating to collect data from all independent DBS studies into one meta-analysis to better understand the performance and limitations of each technology when used with DBS samples. We believe such an analysis will provide a robust understanding of the performance of these technologies and inform guidance and recommendations for country programs considering how to effectively implement the use of DBS to expand access to viral load testing, especially in the wake of the new WHO Consolidated Guidelines recommendation to use viral load testing for monitoring of ART patients.

The objectives of this meta-analysis are to:

- Identify, collate and summarize DBS VL evaluations that have been conducted to date.
- Use combined data stratified on ART-naïve and ART-experienced to assess the analytical performance of DBS compared with plasma on each traditional technology.
- Use combined data stratified on ART-naïve and ART-experienced to assess the clinical utility of DBS for viral load testing, including misclassification levels.
- Assess any differential in the accuracy and precision of DBS by type of sample collected, including venous blood and capillary blood.
- Assess for any potential correction to variability seen when using DBS samples for viral load testing.
- Provide recommendations for the appropriate use of DBS samples for viral load testing, including required training, quality assurance, software adjustments, etc.

Data safety - We would like to include your study in our analysis that will use observation level data. Unique identifier numbers should be used instead of any patient names, numbers or other identifiers that can be linked to the patient. Any country or patient information will be de-linked in the analysis. Original data will be kept strictly confidential and not be shared outside of the

meta-analysis research team. Following completion of the study, data can be deleted from all study files if requested at the end of the study. The study is expected to end before December 2014. A data sharing agreement is also provided with this letter and will be signed by the study leads and you.

Acknowledgement and assurance - We will give credit to all principal investigators who contribute data to this meta-analysis through co-authorship within an International DBS for VL Diagnostics Investigation Consortium, which will be included in any and all publications related to this study. Further, prior to dissemination through publication or other format, all PIs will receive a draft of the methods used and findings and have the opportunity to review and comment on the results, and withdraw their cohort from the meta-analysis if they choose.

Please let us know if you are able to include your data in this valuable research that will help to provide guidance and recommendations on use of DBS samples for viral load testing, or if you have any further questions. We would like to set a deadline for study participation and data inclusion for November 1st, 2013.

Sincerely,

Team member

Template of the Data Sharing Agreement for Dried Blood Spot Samples for Viral Load Testing Meta-Analysis Study Data sharing. A similar letter will be sent to principal investigators using dried plasma spots.

Data Sharing Agreement for Dried Blood Spot Samples for Viral Load Testing Meta-Analysis Study Data sharing

This signed document between ______________________________ *("DBS for VL Diagnostics Investigation Consortium/Study Organization")* and _______________________________ *(Meta-Analysis Study Collaborator)* signifies agreement in data sharing protocol that will be adhered to by all participating parties by the following guidelines to ensure patient, data and study safety:

- Data will not be shared beyond the Meta-analysis study leads at the United States CDC (CDC), NHLS CHAI and LSHTM.
- Patient names and/or patient ID numbers should not be included in data sets to be shared. Unique identifiers should be used to identify observations. If data is received with either patient names or patient ID numbers, these will be replaced by unique identifier numbers and names and IDs will be removed.
- Data may be encrypted if requested by either party.
- Country-specific data will be de-linked if necessary and requested.
- Data will not be used outside of the DBS VL meta-analysis study for any other purpose or analysis.
- Data may be deleted from all meta-analysis cohort computers and any hard copy documents will be destroyed upon completion of the study which will be by the end of December 2014.

DBS VL study contributor	*Meta-analysis study team member*
Date	*Date*
Organization	*Organization*
Title	*Title*

Below is a preliminary list of the studies to date comparing DBS samples to plasma for HIV viral load testing.

Abbott RealTime HIV-1

- Abravaya *et al*, IAS 2008
- Arredondo *et al*, JCM 2012
- Garrido *et al*, JCM 2009
- Lofgren *et al*, AIDS 2009
- Marconi *et al,* CMI 2009
- Mbida *et al*, JAIDS 2009
- Mwau *et al*, manuscript in preparation
- Vidya *et al,* JVM 2012

bioMerieux NucliSENS EasyQ HIV-1

- Abdo *et al*, CROI 2008
- Alvarez-Munoz *et al*, AMR 2005
- Ayele *et al*, JCM 2007
- Brambilla *et al*, JCM 2003
- Delaugerre *et al*, manuscript in preparation
- Fiscus *et al*, JCM 1998
- Garrido *et al*, JCM 2009
- Johannessen *et al*, CID 2009
- Kane *et al*, JVM 2008
- Leelawiwat, JVM 2009
- Lira *et al*, AV 2010
- Metcalf *et al*, CROI 2013
- Monleau *et al*, CROI 2012
- Mwaba *et al,* Lancet
- Ngwende *et al*, IAS 2012
- Rottinghaus *et al*, CID 2012
- Uttayamakul *et al*, JVM 2005
- Van Deursen, JCV 2010

Roche COBAS AmpliPrep/TaqMan HIV-1

- Aitken *et al*, JCM 2013
- Andreotti *et al*, JVM 2010
- Brambilla *et al*, JCM 2003
- Carmona *et al*, IAS 2011
- Delaugerre *et al*, manuscript in preparation
- Ikomey *et al*, JIAPAC 2009
- Mwau *et al*, manuscript in preparation
- Okonji *et al*, JVM 2012

- Ouma *et al*, JCM 2013
- Reigadas *et al*, JVM 2009
- Sekesai *et al*, IAS 2013
- Waters *et al*, JAIDS 2007

Siemens VERSANT kPCR Molecular System

- Pirillo *et* al, JAC 2011
